# Understanding the electronic pi-system of 2D covalent organic frameworks with Wannier functions

**DOI:** 10.1038/s41598-023-28285-w

**Published:** 2023-01-30

**Authors:** Konrad Merkel, Johannes Greiner, Frank Ortmann

**Affiliations:** TUM School of Natural Sciences, Technical University of Munich, Munich, Germany

**Keywords:** Theoretical chemistry, Electronic properties and materials

## Abstract

We investigate a family of hexagonal 2D covalent organic frameworks (COFs) with phenyl and biphenyl spacer units and different chemical linker species. Chemical trends are elucidated and attributed to microscopic properties of the $\pi$-electron-system spanned by atomic $p_z$-orbitals. We systematically investigate the electronic structure, delocalization of electronic states, effects of disorder, bond torsion, and doping, and correlate these with variable $\pi$-conjugation and nucleus-independent chemical shift (NICS) aromaticity. Molecular orbitals are obtained from maximally localized Wannier functions that have $\sigma$- and $\pi$-character, forming distinct $\sigma$- and $\pi$-bands for all valence states. The Wannier-orbital description goes beyond simple tight-binding models and enables a detailed understanding of the electronic topology, effective electronic coupling and delocalization. It is shown that a meaningful comparison between COFs with different chemical elements can only be made by examining the entire $\pi$-electron system, while a comparison of individual bands (e.g., bands near the Fermi energy) can be a insufficient to derive general design rules for linker and spacer monomer selection. We further identify delocalized states that are spread across tens or hundreds of pores of the 2D COFs and analyze their robustness against structural and energetic disorders like out-of-plane rotations of molecular fragments, different strength of energetic disorder and energetic shifts due to chemical doping.

## Introduction

Layered 2D COFs^1–3^ are attracting huge scientific interest because they combine different worlds, namely the construction paradigm of covalently linked molecular building blocks such as in linear polymers with the intriguing features of inorganic 2D materials such as graphene or transition-metal dichalcogenides. After pioneering synthetic works demonstrating the feasibility of 2D COFs^4^ and development of advanced synthetic methodologies^5–7^ for tuning crystallinity, pore size or surface area^8–10^, the research directions for 2D COFs have diversified greatly^11–13^, where the electronic properties are a common theme. Recent improvements in material quality^14^, have triggered the quest for unveiling the intrinsic electronic properties of 2D COFs^15^. Moreover, the active control over these by synthetical strategies would be desirable. This requires a local monomer-based understanding of the emergence of these properties that includes the type of linkage between the monomers^1–3^ which in itself controls the electronic coupling and macroscopic observables such as charge transport parameters. To rationalize the influence of both building blocks and their electronic coupling, one uses effective lattice models^16–19^ for specific electronic bands to explain intriguing band features, which however, are not well-connected to the molecular building units used by synthetic chemists and not to the linkage chemistry.

From a chemist’s perspective, these electronic properties are rooted in the $\pi$-conjugation^1,20^, which is also associated with thermal and chemical stability. This concept is one of the most-frequently cited ones in 2D COF research. For semiconducting 2D COFs, $\pi$-conjugation is frequently connected with improved electronic properties, delocalization of states and good charge-carrier transport, but this connection is usually not supported or quantified and hence remains elusive. While for single molecules a number of different measures for $\pi$-conjugation or aromaticity, that quantify the entire $\pi$-electron system, have been suggested, e.g. resonance energy^21,22^, aromatic stabilization energy^23–25^, multicenter bond index^26,27^, Bird index^28^, Fluctuation index^29^, Shannon aromaticity^30^ or nucleus independent chemical shift (NICS)^31–34^, and are routinely simulated for monomers, this has not been done for 2D COFs. Notwithstanding, it is expected that also for 2D COFs, $\pi$-conjugation is an important prerequisite for dispersive electronic bands^35^, low band gaps, low effective masses^36^ and that it affects optical properties or allows for high carrier mobilities^37^. In this sense, the route towards better $\pi$-conjugated systems is believed to be an important design principle^38^ provoking claims of “high $\pi$-conjugation” or “full $\pi$-conjugation” in recent literature. However, only scant attention has been paid to investigate these measures for 2D COFs and assess their suitability in rationalizing the global electronic structure or to understand delocalization of electrons in these materials, which is necessary for engineering their properties by synthetic approaches.

**Figure 1:**
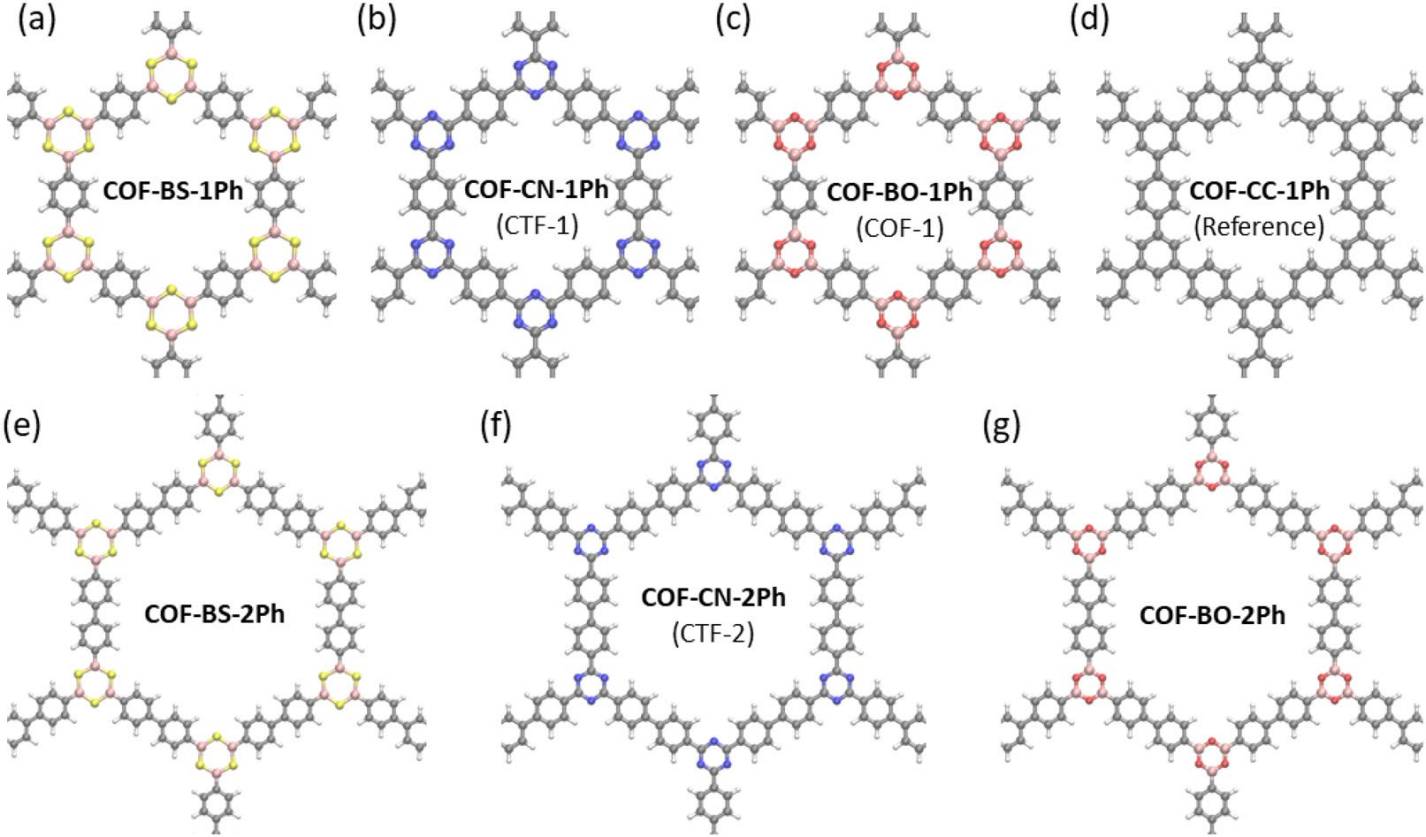
2D COF structures with different linkers and phenyl (**a**–**d**) or biphenyl spacing units (**e**–**g**). Structure (**d**) serves as an idealized reference system for comparison. Chemical elements are represented with different colors: boron (pink), carbon (gray), hydrogen (white), nitrogen (blue), oxygen (red) and sulfur (yellow).

Here we investigate a family of prototypical hexagonal 2D COFs with aromatic and non-aromatic linker molecules in a combined ab initio and model study. The role of the $\pi$-orbitals for their electronic structure and the tendency to form delocalized $\pi$-systems, is analyzed with regard to the chemical nature of the linker unit. Toward this end, localized Wannier orbitals (WO) from first-principles DFT calculations are used to establish topological electronic lattice models. These *WO lattice models* have the advantage over mere *effective lattice models* (representing only few bands) that they are complete and contain all information about the system, including the band entanglement. They also resemble the Lewis structure closely, i.e. they are close to chemical intuition and are thus preferable and universally applicable to any 2D COF. Two manifestations of global $\pi$-conjugation are found, namely the effective coupling of $\pi$-orbitals leading to a large cumulated electronic bandwidth and the electron delocalization over linker and spacer units. Both properties are in general independent of each other and show different chemical trends. We show that whereas aromatic linkers may exhibit flat bands indicating insulating electrical characteristics, 2D COFs with non-aromatic and very polarized linkers in contrast can have a delocalized, global $\pi$-system, challenging the wisdom that formally better $\pi$-conjugation would be sufficient or necessary for better transport properties or delocalization of states.

## Results and discussion

### 2D COF systems and NICS aromaticity

To investigate the in-plane $\pi$-conjugation, we choose seven 2D COFs in monolayer geometry with different linker molecules, which are shown in Fig. 1. All structures are fully planar and belong to the P6/mmm space group, while the sheet separation is chosen sufficiently large to avoid any interaction. For clarity, all materials are named according to their linker elements and number of phenyl rings in the spacer unit (see Fig. 1). Common aliases, if they exist, are provided in parentheses. Among the systems, COF-CC-1Ph (Fig. 1d) is included as a hydrocarbon reference system, whose phenyl linker has no polarity in its carbon-carbon bonds and potentially leads to optimized conditions for a delocalized $\pi$-system. We note that, despite obvious steric effects, also COF-CC-1Ph is studied in its planar configuration because our focus is on the comparison of the electronic properties of the seven 2D COFs in the first place, while bond torsions will be studied further below. On the other hand, COF-BO-1Ph^4^ and COF-BO-2Ph^39,40^ with their strongly polarized BO bonds are conventionally considered to be only weakly conjugated, if at all (*vide infra*), thus representing the opposite case in our series of test systems with variable electronic properties. The triazine linker in COF-CN-1Ph^5^ and COF-CN-2Ph^41^ has less strongly polarized bonds and may be considered an intermediate case. The comparision between carbon-based and boron-based linkers enables us to distinguish between aromatic and non-aromatic linker monomers (*vide infra*). BS-based linkers add another interesting variant to the family studied here. The electronic structure and wave functions of these systems are described by density functional theory (DFT)^42^ for the relaxed structures (details are provided in the “Methods” section).

**Figure 2:**
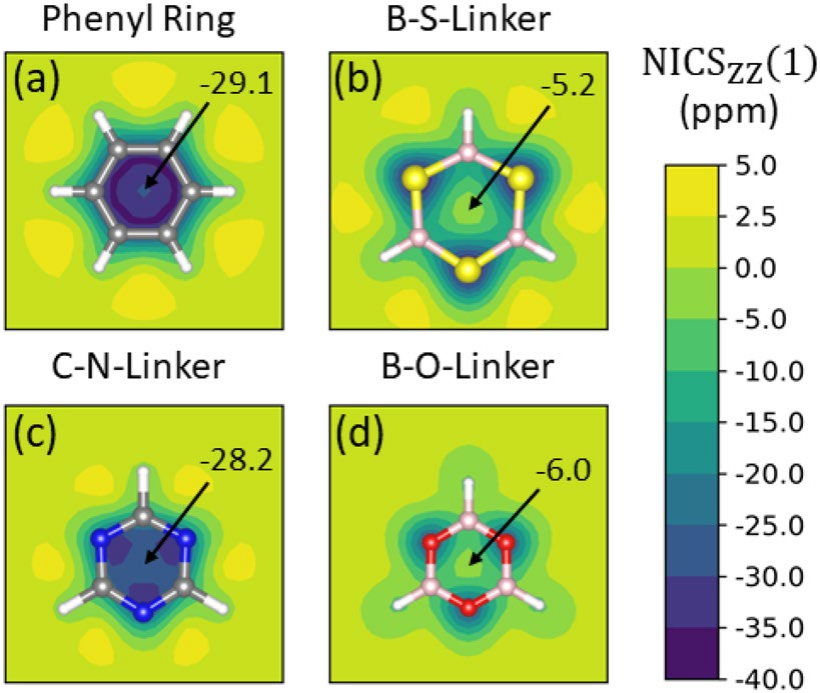
$\text {NICS}_{ZZ}(1)$-scan for all basic building blocks of the investigated COFs. Linker units are terminated with hydrogen.

To characterize $\pi$-conjugation of the studied 2D COF systems, we calculate NICS, which is a well-established measure of aromaticity^31–34^. It is, in principle, applicable to macrocyclic systems and even to non-planar structures^43–45^, which is a major advantage over other measures of aromaticity that are often applicable only to single molecules, single rings or rely on suitable non-aromatic reference systems. NICS measures the response to an external magnetic field in an NMR Gedanken experiment, where a ring current is induced in cyclically conjugated molecules, which in turn causes a magnetic field that counteracts and therefore shields the external field. The $\text {NICS}_{ZZ}$ value is defined as the negative ZZ-component of the shielding tensor (out-of-plane direction) and is usually evaluated at the center or ${1}\,{\text{\AA} }$ above the center of a cyclic molecule. A more comprehensive picture is obtained by performing NICS scans across the molecular planes^46,47^.

**Figure 3:**
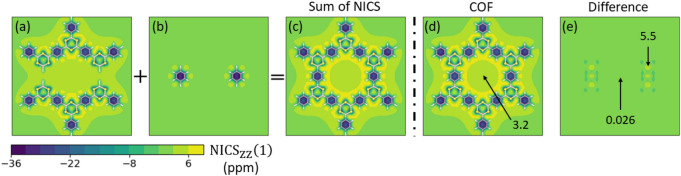
$\text {NICS}_{ZZ}(1)$-scans (${1}\,{\text{\AA} }$ above the molecular plane) for a single pore of COF-BS-1Ph. (**a**–**c**) The decomposition scheme that is compared with calculations of the entire pore (**d**). All other investigated COFs show similar results.

Before we investigate NICS values for the COF pores, we start by examining the basic building blocks, i.e. the linker and phenyl rings. Figure 2 shows their $\text {NICS}_{ZZ}(1)$ values. Strongly negative values in the middle of the ring indicate aromatic molecules^33,34^ as seen for phenyl- and CN-linker rings. As compared to the most aromatic monomers, both boron containing linkers show drastically reduced absolute but still non-zero $\text {NICS}_{ZZ}(1)$ values at the center. We note that they stem from sulfur or oxygen atoms and cannot be associated to aromatic ring currents. These linker units are therefore reminiscent of non-aromatic species. The NICS plots also show that the ring currents not only generate a magnetic field inside the rings, but also outside. There the direction of the magnetic field is reversed as described by Ampères law. This leads to a shielding of the external magnetic field inside the rings but amplifies the external magnetic field outside the rings in the molecular plane, as is clearly visible for all monomers. Such outfield effects are very important for a sound interpretation of NICS values of multiple ring systems such as anthracene^48,49^. In these systems, multiple ring currents around every single ring (and every combination of rings) can occur. Magnetic fields generated from one ring current also influence neighboring rings and shift the NICS value to positive values^34,46,50^.

In order to address this question for 2D COFs, different contributions from all rings need to be analyzed. Our study here focuses on the induced ring current of a single 2D COF pore rather than all kinds of local ring currents, because we are interested in the characterization of global $\pi$-conjugation for 2D COFs and not local $\pi$-conjugation of single monomer units. Therefore, we need to distinguish the NICS values originating from ring currents around the COF pore (that are of interest) from all other sources of NICS originating from the peripheral building blocks. Towards this end, we split the COF pore into fragments as shown in Fig. 3a,b such that no ring current can flow analogously to an open electrical circuit. NICS values for this situation of a broken conjugation are compared to a completely closed COF pore, where ring currents are possible (Fig. 3d). The COF fragments in Fig. 3a,b are saturated with additional hydrogen atoms and the NICS maps resemble those of the monomers in Fig. 2. The NICS values of all fragments are added in Fig. 3c. Interestingly, this yields values very similar to the ones of a completely closed 2D COF pore (Fig. 3d) although the fragmented geometry prohibits a pore ring current whereas the closed geometry might support such current. For better analysis, Fig. 3e shows the difference in the NICS values ($\Delta$NICS) between the sum of the COF fragments and the full COF pore. This $\Delta$NICS plot confirms that the difference vanishes. Only minor contributions that are due to the breaking of the chemical bonds at local points remain where the two phenyl rings were cut out, which is a purely local effect that is not associated to the COF pore. The same results were found for calculations of all other investigated systems. Therefore, the NICS values of the investigated COFs can be understood as the superposition of NICS values of the smaller peripheral rings surrounding the pore. Possible contributions from *pure pore-ring currents*, which would manifest in a finite $\Delta$NICS value in Fig. 3e, are found to vanish in any of the COFs up to numerical precision of the simulations. We emphasize that several tests of more elaborate decomposition schemes for the COFs did not give measurable $\Delta \text {NICS}$ values from ring currents for any of the investigated systems (see section SI-8 in the Supporting Information).

This leads us to the conclusion that, despite clear signatures of NICS aromaticity of the different linkers, NICS calculations for an entire 2D COF are only of limited use. They cannot provide insight into the role of $\pi$-conjugation-induced formation of extended states that possibly could extend over many pores. We therefore study other measures that are more suitable here subsequently.

### Orbitals and band structure

We next analyze the DFT electronic structure, which is fully described by the Bloch states $|m {\varvec{k}}\rangle$ and the Kohn–Sham Hamiltonians ${\hat{H}} = \sum _{m{\textbf{k}}} \epsilon _{m}({\textbf{k}}) {\hat{a}}^\dagger _{m{\textbf{k}}} {\hat{a}}_{m{\textbf{k}}}$. For a better analysis of structural and energetic disorders, which are rather intransparent in k-space, we represent the Kohn–Sham states with localized orbitals that are associated to linker or phenyl/biphenyl spacer moieties. We choose WOs in the concrete form of maximally localized Wannier functions (MLWF)^51^, which are particularly well suited since they provide a natural way to obtain localized orbitals for periodic crystals (see “Methods” section for all details). It has been shown that if all occupied states (valence bands) are contained in the Wannierization procedure, MLWF reproduce typical bonding orbitals such as sp3-hybrid orbitals in Si and GaAs and $\sigma$- and $\pi$-orbitals in hydrocarbons^51,52^. They are obtained from the Bloch states by a unitary transformation according to

1 $$\begin{aligned} |n {\varvec{R}}\rangle = \sum _{m k} e^{i {\varvec{k}}{\varvec{R}}} U_{n m}({\varvec{k}}) |m {\varvec{k}}\rangle , \end{aligned}$$

and therefore retain all electronic information about the system. ${\varvec{R}}$ indicates the unit cell associated to the WO and the unitary matrix $U({\varvec{k}})$ can be chosen such that the obtained orbitals have minimum spread^51,53^. For this reason MLWF are commonly used for band structure interpolation^54^ and calculations of topological properties, e.g. Berry phases or Chern numbers^55^. The transformed Kohn–Sham–Hamiltonian in the WO basis reads

2 $$\begin{aligned} {\hat{H}} = \sum _{ij} \epsilon _{ij} {\hat{a}}^\dagger _i {\hat{a}}_j, \end{aligned}$$

where ${\hat{a}}^{(\dagger )}_i$ (create) annihilate an electron at the *i*-th orbital, $\epsilon _{ii}$ are the orbital (onsite) energies and $\epsilon _{ij}$ with $i\ne j$ are transfer integrals (TI). We have checked that the transformed Hamiltonian Eq. (2) accurately reproduces the Kohn–Sham electronic structure (see Fig. SI-1 in the Supporting Information).

**Figure 4:**
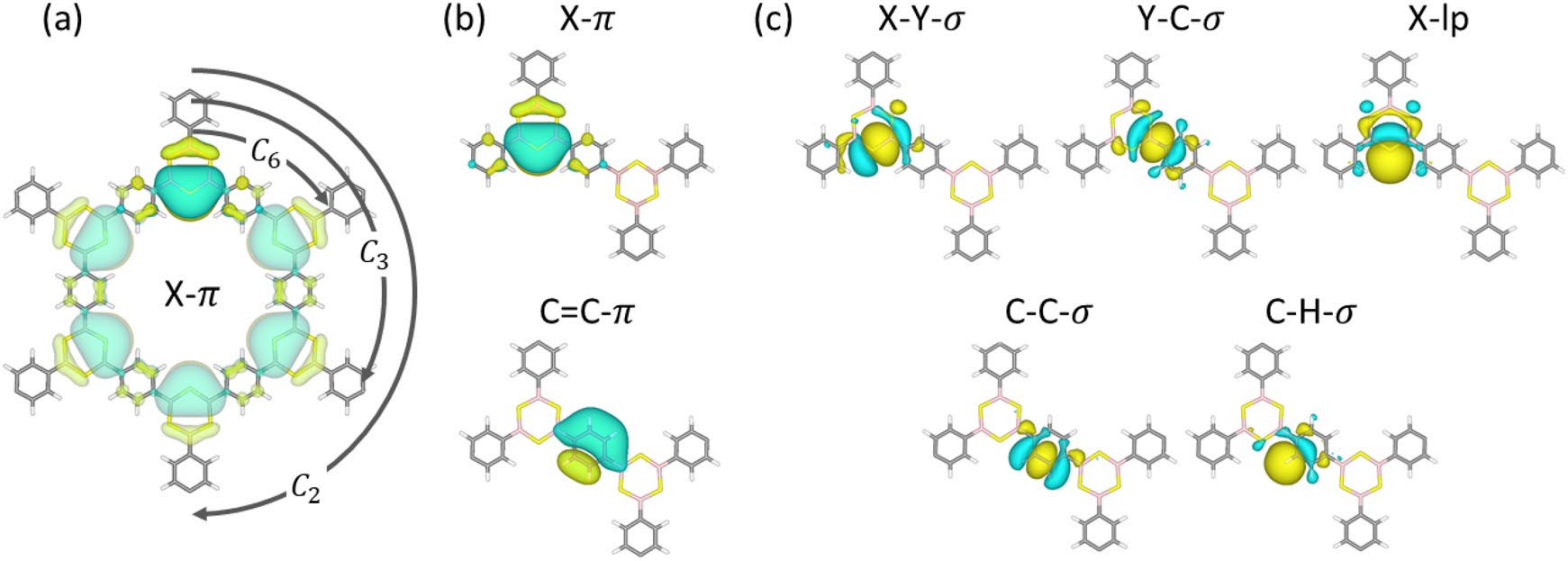
Illustration of Wannier orbitals obtained for all materials. (**a**) The symmetry equivalent WO of the type X-$\pi$. (**b**,**c**) All different bond-types in terms of WO at linker and phenyl rings. The notation of WO is based on the atom types (X,Y,C,H) and shapes ($\pi$, $\sigma$, lp). All studied COFs contain the same shapes of WO.

Figure 4 shows the resulting WOs for COF-BS-1Ph as example representing all 2D COF structures in this work. We observe that all WOs are localized within the range of $\sqrt{ \langle x^2 \rangle - \langle x \rangle ^2}\le {1.4}\,{\text{\AA} }$ which is close the C=C bond distance. They can be associated with typical bonding hybrid orbitals. As an example, Fig. 4a shows the WOs of the type X-$\pi$ (X = S, N, O), which are localized at a single linker moiety (e.g. the uppermost BS linker with its WO in darker colors). It also shows the copies of the orbital at the six symmetry equivalent positions in the COF. In addition to these six X-$\pi$ orbitals, Fig. 4b,c illustrates a complete set of all occurring types of WOs at linker and spacer units. Although WOs of the same type share the same shape, small deformations in the vicinity of the linker can occur and are shown in Figs. SI-2 and SI-3.

Similar to the X-$\pi$ WOs at the linker, $\pi$-orbitals of type C=C-$\pi$ are located at the phenyl rings (three $\pi$-orbitals per ring). The $\pi$-orbitals at linker and phenyl rings together form the $\pi$-system (Fig. 4b), which contains in total 15 $\pi$-orbitals per unit cell for COF-BS-1Ph, COF-CN-1Ph, COF-BO-1Ph and COF-CC-1Ph (Fig. 1a–d) and 24 $\pi$-orbitals per unit cell for COF-BS-2Ph, COF-CN-2Ph and COF-BO-2Ph (Fig. 1e–g). These are by symmetry the only WOs which have contributions from atomic $p_z$-orbitals.

Figure 4c shows all $\sigma$-orbitals (apart from symmetry equivalent copies) that represent the bonds between the chemical elements (type X-Y-$\sigma$, for which X = S, N, O, C and Y = B, C are the linker atoms). At every phenyl and linker ring there are six WOs of type C-C-$\sigma$ or X-Y-$\sigma$, respectively. Together with the C=C-$\pi$ and X-$\pi$ orbitals we find a typical structure of alternating single and double bonds that is in agreement with the Lewis structure for all COFs. The C-C-$\sigma$ orbitals at single-bond and double-bond positions have the same shape. However, they differ in other properties and, if necessary, we distinguish these two sub-types in our notation as C-C-$\sigma _s$ and C-C-$\sigma _d$, respectively. In addition, lone-pair orbitals at the linker are denoted as X-lp (X = S, N, O). Note that, since COF-CC-1Ph only consists of phenyl rings as building blocks, it does not host X-$\pi$ or X-lp orbitals but C=C-$\pi$ and C-H-$\sigma$ orbitals instead. In total one obtains 69 WOs per unit cell for COF-BS-1Ph, COF-CN-1Ph, COF-BO-1Ph and COF-CC-1Ph and 111 WOs per unit cell for COF-BS-2Ph, COF-CN-2Ph and COF-BO-2Ph.

Having established the WOs as convenient basis of our study, we turn to their electronic coupling and possible formation of globally extended states and their robustness. The distribution of such states over linker and phenyl rings, spanning over several pores or even throughout the entire 2D COF, would indicate interesting electronic and transport properties. This requires the electronic coupling between adjacent orbitals, while their energies should not differ too much.

### Band structure, bandwidth, and lattice models

In order to investigate the influence of $\pi$-orbitals on the electronic structure, we analyze the $\pi$-system as a whole rather than restricting ourselves to specific bands, because a preselection of a subset of bands could be misleading. Toward this end, we determine the contribution of the $\pi$-orbitals to the Kohn–Sham-eigenstates $| n {\varvec{k}}\rangle$ by projecting them onto all $\pi$-orbitals in a unit cell according to the weight

3 $$\begin{aligned} P_\pi (n,{\varvec{k}}) := \sum _{i\in \pi \text {-WOs}} |\langle i | n {\varvec{k}}\rangle |^2. \end{aligned}$$

**Figure 5:**
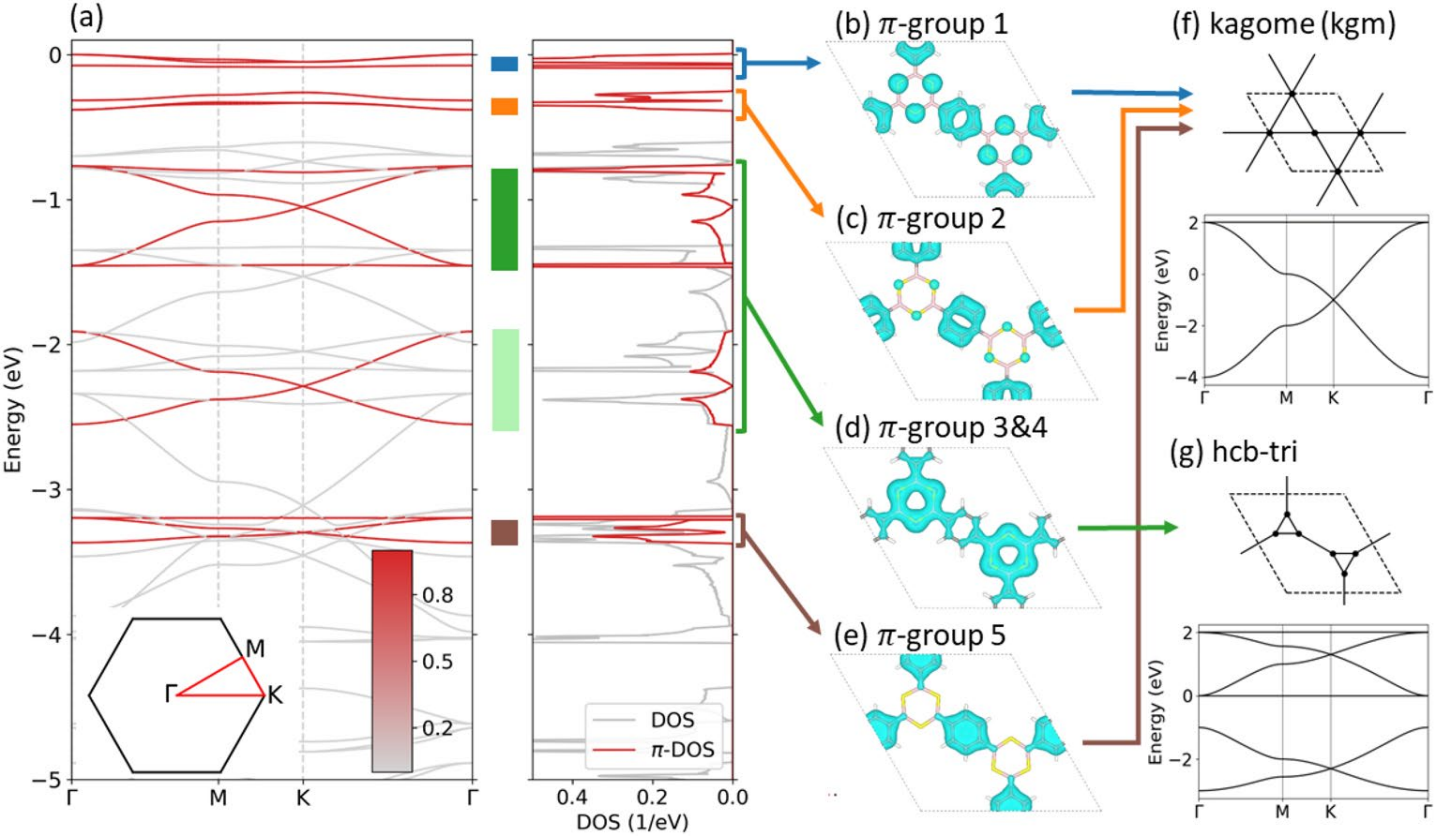
(**a**) Band structure of COF-BS-1Ph, projected on all $\pi$-orbitals, and density of states (DOS) for all states and $\pi$-states ($\pi$-DOS). Zero energy is put to the top of the valence bands. Colored stripes indicate the bandwidth for every group of $\pi$-bands. The Brillouin zone is shown as inset. (**b**–**e**) Partial charge densities at the $\Gamma$-point from the indicated group of $\pi$-bands, $|\sum _{n} \langle n{\varvec{k}}=0 | \psi \rangle |^2$ (where *n* is restricted to a single $\pi$-group). (**f**,**g**) Comparison to the 2D effective lattice models.

Figure 5a shows the electronic structure for the uppermost valence states of COF-BS-1Ph and the projection $P_\pi (n,{\varvec{k}})$ (red color bar) together with the density of states (DOS) and the $\pi$-projected DOS ($\pi$-DOS) for COF-BS-1Ph. Band structures for all other COFs can be found in section SI-3 of the Supporting Information.

The $\pi$-orbitals only contribute to a specific subset of bands, subsequently denoted $\pi$-bands. Protected by symmetry, these $\pi$-bands are decoupled from all other bands, i.e. they do not have contributions from $\sigma$- or lone-pair-orbitals and vice versa, allowing a clear distinction of the $\pi$-system from all other bands. Although, the $\sigma$- and lone-pair-bands differ strongly between different COFs, we see that the $\pi$-system is very similar in shape and arrangement of the bands. Only 2Ph-COFs host additional $\pi$-bands, however, similarities between 1Ph and 2Ph COFs are still obvious. We can therefore conclude that the qualitative energy dispersion is mainly determined by the geometry and symmetry of the COF, whereas the chemical elements of the linker manifest themselves solely in the bandwidth of individual $\pi$-groups.

We briefly discuss the origin of these $\pi$-groups using COF-BS-1Ph as an example. For convenience of presentation, we indicate in Fig. 5a the different groups with colored stripes, which will be used throughout this paper. The first two $\pi$-groups (blue and orange) are distorted kagome (kgm) bands (cf. Fig. 5f for a kgm lattice and its idealized band structure)^16–18^. Their partial charge density is mainly localized at typical kgm sites as expected (cf. Fig. 5b,c). A deeper analysis of the Hamiltonian in the WO basis reveals that the distortion is not related to symmetry-breaking but originates from (small) next-nearest neighbour interactions. In agreement with previous studies^56^ these distortions can be reduced by removing corresponding transfer integrals. $\pi$-group 3 and 4 (dark and light green) have the same origin and show the same partial density (cf. Fig. 5d). The corresponding real-space lattice is a honeycomb lattice in which the simple vertex is replaced by a connected trimer (cf. Fig. 5g), which we call “hcb-tri”^18,56^. The fifth group of $\pi$-bands (brown) shows a nearly ideal kgm band structure. In consistency, the corresponding partial density in Fig. 5e is located at the phenyl units, which, at first glance, seems to be a perfect manifestation of a kgm lattice that is realized by phenyl-based $\pi$-WOs (Fig. 5f). The visual identification of an effective lattice model from crystal states, however, can be tricky and caution should be exercised. For instance, in the present case, the Hamiltonian in the WO basis does not contain significant TIs between the WOs at the phenyl rings, in contradiction to the kgm lattice model. The absence of these (through space) TIs between WOs at the phenyl rings is explained by the fact that long range connections between WOs are exponentially suppressed and significant TIs only exist over short distances (few Angstroms). An overview is compiled in Figs. SI-4 and  SI-5 of the Supporting Information. This case exemplifies that it is generally not permitted to infer an effective lattice model just from the band structure. The TIs that are responsible for these kgm-bands are those between WOs of the phenyl units and the BS linkers. The linkers therefore serve as bridging units that facilitate the carriers tunneling from one phenyl ring to the other through the linker (and not through space). As a consequence, the linkers, even if devoid of charge density for these bands, can have a strong impact on these bands (see “Impact of orbital energies” below). This shows that picking isolated groups of bands for an effective lattice model may not capture all its properties and can be a misleading simplification that may not correctly describe the origin and entanglement of the bands, while the formal representation in a Wannier basis is exact.

**Figure 6:**
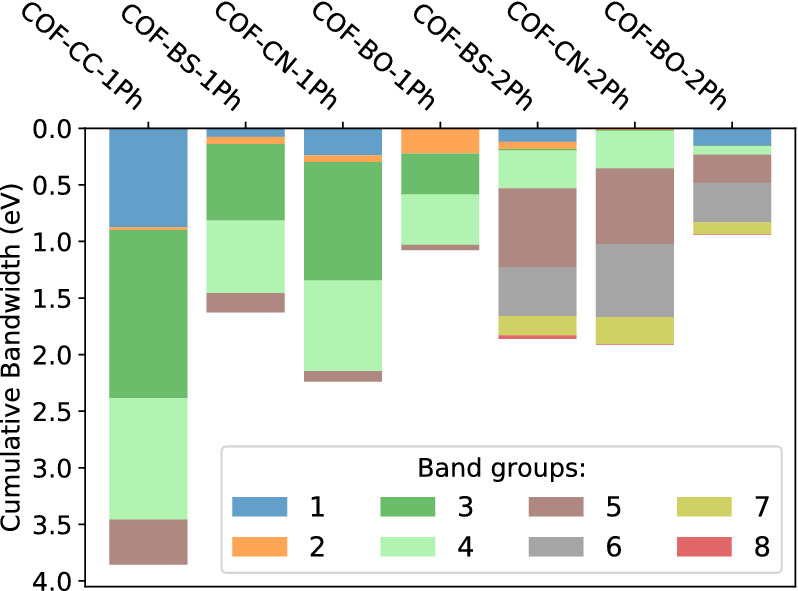
Electronic bandwidths for planar structures. Colors represent different groups of bands as highlighted in Fig. 5 for COF-BS-1Ph. The overall size of the bars indicate the cumulative bandwidth. Values are provided in Tables SI-1 and SI-2.

The $\pi$-system is analyzed further by quantifying its electronic bandwidth. Our focus is on the cumulated bandwidth of the entire $\pi$-system in Fig. 6, while the individual contributions are also resolved in the figure. From comparing the different COF structures, we find the cumulative bandwidths to depend sensitively on the linker type. We observe similar trends for phenyl (1Ph) and biphenyl (2Ph) cases. The reference system COF-CC-1Ph has the largest cumulative bandwidth, which confirms the conjecture of the highest degree of (global) conjugation for this material. The two boron-based COFs have much lower values. The weaker $\pi$-conjugation in presence of boron atoms leads to a reduction in the cumulated bandwidth by about a factor of two in case of BS COFs and by a factor of about three to four in case of BO COFs. This reduction is in full consistency with the combination of boron, an electron deficient atom with electron-rich oxygen or sulfur. Also, COF-BO-1Ph and COF-BO-2Ph are the COFs with the most polarized linker according to the electronegativity values of the chemical elements and Bader charges (see section SI-4 in the SI).

In contrast to the cumulated bandwidths, these chemical trends are not reflected in the top valence bands. These bands, which are responsible for (p-type) transport properties, are $\pi$-bands for almost all investigated systems except for COF-CN-1Ph, where the top valence bands originate from lone-pair orbitals. The widths of these bands do not follow the same trend as the cumulative bandwidth, which makes transport parameters like the effective mass (see Table SI-4 of the SI) uncorrelated to the overall $\pi$-conjugation, which is a rather unexpected finding. Furthermore, the top valence bands are often kgm groups which contain a flat band that yields huge effective masses (see also^56^). For transport properties it is therefore crucial if the flat band is on top or below the dispersive bands, which can even change for different COFs with the same linker, e.g. in the case of COF-BO-1Ph and COF-BO-2Ph.

### Impact of orbital energies

**Figure 7:**
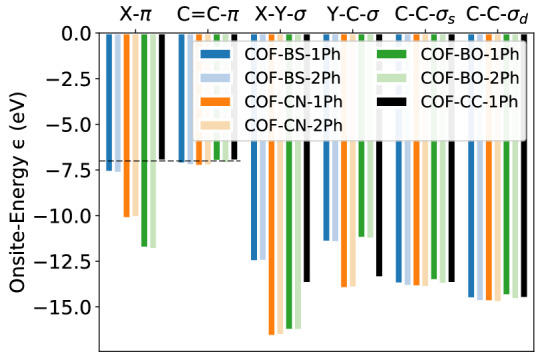
Orbital onsite energies for every WO and material. C-C-$\sigma _s$ and C-C-$\sigma _d$ denote the C-C-$\sigma$ orbitals at single and double bond positions, respectively.

Orbital energies are key electronic parameters for many applications. They determine redox potentials of the monomers, which can be used to control the COF’s electronic properties. They can be further tuned by chemical doping, which is frequently performed for 2D COFs^37,57–60^. In addition, orbital energy differences have a strong impact on the band structure. For instance, they can lead to a reduction of the band width and to a gap opening in the bands. A prominent example for the latter is the difference between the atomic 2D crystals graphene vs. hexagonal BN. In 2D COFs, owing to the symmetry, WOs that belong to the same bond-type for a given COF share the same orbital energy, while differences occur for C-C-$\sigma _s$ and C-C-$\sigma _d$ at single and double bond positions, respectively. In contrast, the orbital energies of analogous WOs in different 2D COFs vary substantially. Figure 7 compares the WO orbital energies for all systems. Colors represent different linkers and their pale version represent the biphenyl COFs.

The C=C-$\pi$, C-C-$\sigma _{s}$ and C-C-$\sigma _{d}$ orbitals at the phenyl rings have, respectively, similar onsite energies for all COFs, including the reference system COF-CC-1Ph, with variations within a range of maximally ${0.37}\,{\hbox {eV}}$ among each individual group, while all other orbital energies depend much more strongly on the chemical species. Indeed, the onsite energies of the linker orbitals ($\epsilon _{\text {S-}\pi }$,$\epsilon _{\text {N-}\pi }$, $\epsilon _{\text {O-}\pi }$) show big differences depending on their chemical elements and the polarization of the bond, ranging between ${-11.85}\,{\hbox {eV}}$ and ${-7.63}\,{\hbox {eV}}$.

**Figure 8:**
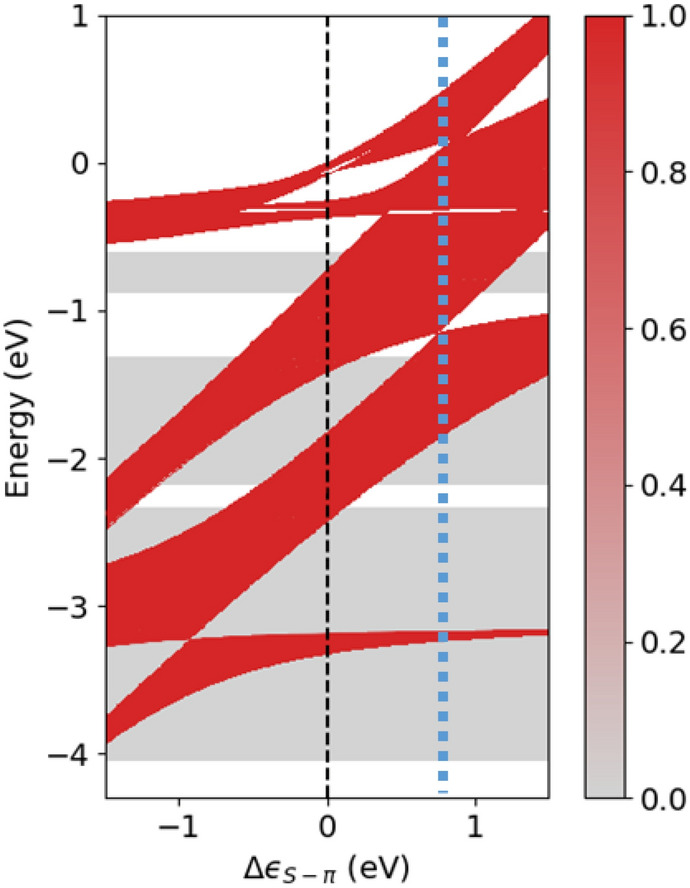
Impact of S-$\pi$ onsite energy on the band structure of COF-BS-1Ph. Red color indicates high values of projection $P_\pi$ onto $\pi$-orbitals. The black dashed line shows the original onsite energy and the blue dashed line highlights the special case where all upper $\pi$-groups touch each other. The energy scale is set to zero at the highest occupied state of the original 2D COF for better comparison.

In order to study how the different WO energies influence the electronic structure, we focus on the X-$\pi$ orbitals at the linkers, which are material dependent as discussed above and have the strongest effect on the $\pi$-system. COF-BS-1Ph is studied as a concrete representative case. It is instructive to study modifications in the WO energy by a simple model according to the replacement $\epsilon _{\text {S-}\pi }\rightarrow \epsilon _{\text {S-}\pi }+\Delta \epsilon _{\text {S-}\pi }$ while all other electronic parameters are kept fixed for the sake of transparency of the effects. Positive values for $\Delta \epsilon _{\text {S-}\pi }$ would correspond to *p*-type doping and could be realized by intercalation of strong electron acceptors such as F4TCNQ^59^. Figure 8 shows the changes in the band structure as a function of $\Delta \epsilon _{\text {S-}\pi }$. For clarity of presentation, the color scale in the figure indicates the maximal projection $P_\pi (E) = \max \nolimits _{{\varvec{k}}\in \Lambda } \{P_\pi (n,{\varvec{k}})\, |\, E_{n}({\varvec{k}})=E\}$ ($\Lambda$ is the path along $\Gamma$-M-K-$\Gamma$ in the BZ) for a given energy *E*. This clearly distinguishes all $\pi$-bands from all other bands regardless of the values of $\Delta \epsilon _{\text {S-}\pi }$.

We observe that the shift $\Delta \epsilon _{\text {S-}\pi }$ leads to strong changes for the bands of COF-BS-1Ph for both *n*- and *p*-type doping regimes. This affects the bandwidths and energies of all $\pi$-bands, whereas all other bands remain unchanged. These changes are even qualitative since various crossovers and gap closures and openings are observed. We also find that even those $\pi$-bands, which have no charge density on the linker (S-$\pi$) are affected by the S-$\pi$ onsite-energy change. For instance, the bandwidth of the fifth group of bands (at an energy below ${-3}\,{\hbox {eV}}$) changes significantly with $\Delta \epsilon _{S-\pi }$, despite the fact that the charge density of this kgm-group is completely localized on the phenyl rings without any contributions from the linker (see Fig. 5e). Above, we have pointed out that there are no direct (through space) TIs between any phenyl-based WOs that lead to the kgm-bands as would be expected by a simple model. There are rather many indirect connections with TIs through the linker (see Fig. SI-4). It is these indirect connections that are influenced by the energy variation of the linker and further corroborate the above findings.

Interestingly at $\Delta \epsilon _{\text {S-}\pi }={0.8}\,{\hbox {eV}}$ (blue dashed line in Fig. 8), we observe that four of the five $\pi$-groups of bands join into a single one with a total band width of ${2.4}\,{\hbox {eV}}$, whereas the lowest-energy band remains separated. The upper $\pi$-groups touch each other but do not overlap. Larger shifts of $\Delta \epsilon _{\text {S-}\pi }>{0.9}\,{\hbox {eV}}$ lead to reconstructed $\pi$-groups such that new gaps appear and flat bands change their group affiliation resulting in $\pi$-groups with different topology. More precisely, the upper two kgm-groups become a hcb-tri group and the former hcb-tri group splits into two kgm-groups, i.e. the topological groups get therefore reordered. Figure SI-10 shows three band structures for selected values of $\Delta \epsilon _{\text {S-}\pi }$ at ${0.5}\,{\hbox {eV}}$, ${0.9}\,{\hbox {eV}}$ and ${1.2}\,{\hbox {eV}}$ for the interested reader. These findings show that the entire $\pi$-system is important for a comprehensive understanding of the COF’s electronic structure and investigations of isolated $\pi$-bands may not provide the full picture. Our results further suggest a very rich playground for manipulating band topologies with dopant-induced orbital energies. This is possible even for rather modest energy shifts, which should be accessible with conventional dopant species that have already been used for 2D COFs in the past^60–62^.

### Robustness and breaking of $\pi$-conjugation by bond torsion

**Figure 9:**
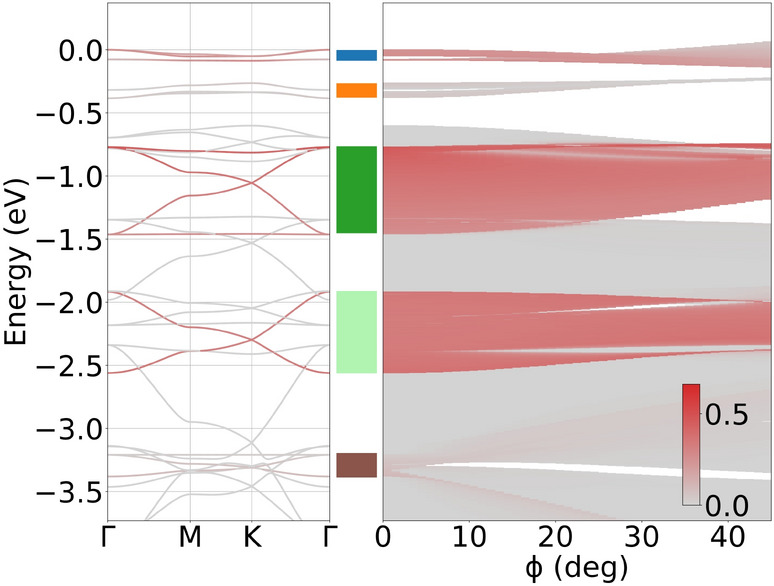
Band structure of COF-BS-1Ph (including projection on $p_z$-orbitals at linker positions) and impact of rotation of the phenyl rings on band width and projection. The energy zero is set to the valence band maximum of the planar structure.

Mechanical distortions like out-of-plane rotations of phenyl rings (around the bonds to their neighbors) may limit the delocalization of electronic Bloch states because they can modify the overlap of $\pi$-orbitals between linker and phenyl rings^63,64^. To investigate these effects on the bandwidth, we rotate all phenyl rings of the 2D COF by the same angle $\phi$ in a propeller like arrangement (P3 symmetry group) while keeping the linker positions fixed. Figure 9 shows the impact of such rotations on the bandwidth for COF-BS-1Ph. Complementary figures for the other structures can be found in the SI (Figs. SI-11, SI-12). For reference, the band structure for the planar geometry is reproduced (from Fig. 5) in the left panel of Fig. 9. For a better overview over different states, they are projected onto atomic $p_z$ orbitals at the linker positions and indicated by red color (see “Methods” section for all details). Projections onto these $p_z$-orbitals at the linker are sufficient to characterize the global, delocalized $\pi$-system because there are no $\pi$-states that are solely localized at the linker.

Upon rotation we observe a change in the bandwidth with increasing $\phi$ for all highlighted $\pi$-bands and for $\pi$-band group two (orange bar), indicating that the out-of-plane-rotation reduces the effective coupling within the $\pi$-system as expected. The largest bandwidth reduction is found for the two $\pi$-groups with the largest width (dark and light green), that correspond to the hcb-tri effective model. Despite the reduction of bandwidth, the band structures of these $\pi$-groups maintain the same order for the groups for all $\phi$. This is different to the onsite energy change investigated above which induced band crossovers, suggesting that structural effects have a somewhat weaker impact as compared to energetic effects. The 2D COFs with rotated groups, however, have a reduced symmetry that leads to additional band gaps in Fig. 9. These gaps exist already at small angles $\phi <20^{\circ }$ and become clearly visible for larger rotations. Despite the emergence of gaps, the band structure remains similar to the planar case for almost all $\pi$-groups. Only for the fifth $\pi$-group (brown) we observe fundamental changes of the band structure, where bands get mixed with energetically nearby bands resulting in a complete disappearance of this $\pi$-group. This is not surprising since the (partial) charge density of these $\pi$-group is entirely located at the rotated phenyl rings (see Fig. 5e). Interestingly, the distortion of the first (blue) and second (orange) kgm bands is reset upon rotation and eventually leads to nearly perfect kgm bands for $\phi \ge {50}^{\circ }$.

**Figure 10:**
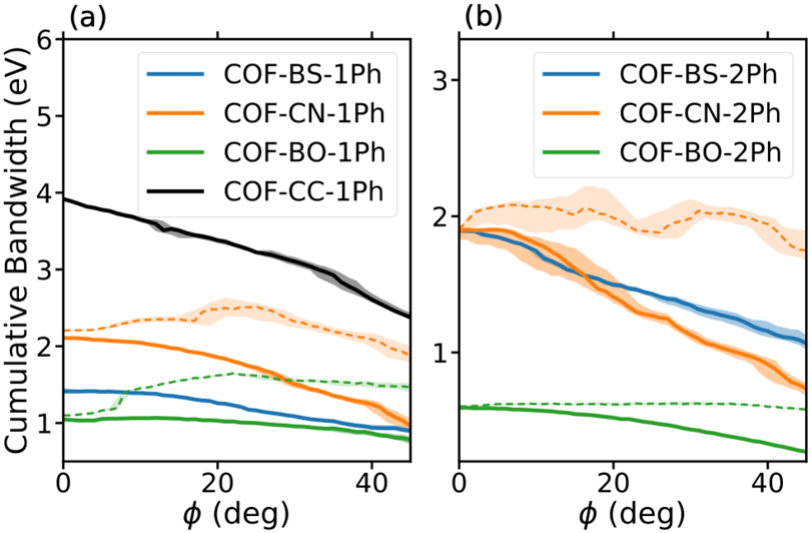
Cumulative bandwidth for different rotation angles $\phi$ for 1Ph COFs (**a**) and 2Ph COFs (**b**). Solid lines focus on the upper $\pi$-groups (near the Fermi-level) and dashed lines show the measurement for all $\pi$-groups. Colored areas indicate the numerical error of CBW due to a change of threshold parameters by 15%.

To quantify the visual impression in Fig. 9, we extract the ($\phi$-dependent) cumulative bandwidth (see Eq. (6) for definition) in Fig. 10 for all phenyl COFs (a) and bi-phenyl COFs (b). Solid lines show the cumulative width of the $\pi$-bands that are already present in the planar systems. We see that the cumulative bandwidth (solid lines) decrease upon rotation for all phenyl COFs. Especially COF-CC-1Ph has not only the largest cumulative bandwidth but also shows the strongest decrease (linear slope). COF-BO-1Ph exhibits only small changes with increasing $\phi$. In the case of 2Ph COFs (b) this trend is less pronounced.

In addition to the reducing bandwidth, in some 2D COFs additional $\pi$-bands appear that do not exist for the flat geometries ($\phi =0$). This occurs at low energies and is accompanied by the complete change of the fifth $\pi$-group (brown). These new bands can have a large impact on the overall cumulative bandwidth which we indicate by dashed lines in Fig. 10.

We finally note that changes of the electronic structure upon rotation can also be evaluated by other quantitative measures which are compiled in the SI in section SI-7.

### Density of states, disorder and delocalization

Of central importance to electronic and transport properties of 2D COFs is the delocalization of the electronic states. While the electronic coupling between $\pi$-orbitals is a central prerequisite, it may not be sufficient to delocalize electronic states beyond the size of individal pores which would be necessary for efficient charge-carrier transport^65,66^.

We start by investigating the stability of the $\pi$-bands against increasing strength of energetic disorder. For this purpose we investigate large samples (supercells of $29\times 29\times 1$ unit cells for 1Ph COFs and $23\times 23\times 1$ for 2Ph COFs) that are constructed from the WO-based Hamiltonian (Eq. 2) and add electronic disorder. We use a generic uncorrelated Anderson model which shifts the onsite energy of every orbital randomly with a value $\Delta \epsilon$ from the interval $[-W/2,W/2]$, where the disorder strength *W* is the width of a box distribution^67^. We calculate wave functions and their energies at the center of the supercell BZ (see “Methods” section).

**Figure 11:**
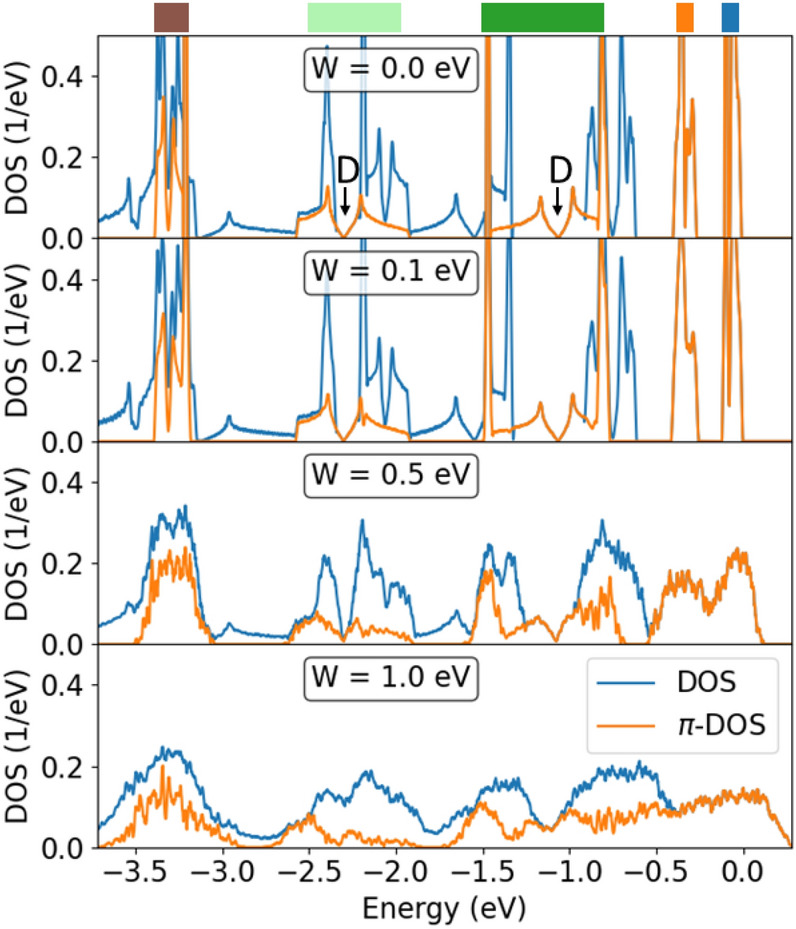
DOS and $\pi$-DOS for COF-BS-1Ph with different amounts of Anderson disorders *W*. Disorder-free case reproduced from Fig. 5a for comparison. Colored bars and energy scale are the same as in Fig. 5. Labels “D” show the Dirac points of the hcb-tri group.

Figure 11 shows the DOS and the $\pi$-DOS of COF-BS-1Ph for different strengths of disorder *W*. $\pi$-groups are clearly distinguishable from all other valence bands and highlighted with colored stripes as before. The Dirac cones in the band structure are clearly visible in the DOS and the $\pi$-DOS with their characteristic V-shapes and are labeled by “D” at ${-1.1}\,{\hbox {eV}}$ and ${-2.3}\,{\hbox {eV}}$. With increasing disorder, these shapes remain visible up to large disorder values ($W={1}\,{\hbox {eV}}$), indicating a good robustness against disorder. Analogous behaviour of the Dirac cone states was observed for graphene, which however has much wider $\pi$-bands^68,69^. The robustness of the states for the more complex 2D COFs with rather moderate bandwidths, however, is quite surprising and suggests a similar mechanism here, which would suggest similar transport phenomena^70^.

In contrast to these features, the two groups of flat $\pi$-bands (at ${0}\,{\hbox {eV}}$ and ${-0.35}\,{\hbox {eV}}$), which we identified as deformed kgm bands, are much more strongly affected by the disorder. These bands are broadened with increasing *W* and the individual DOS peaks of both bands overlap already for $W={0.5}\,{\hbox {eV}}$ and finally merge into a broad feature at $W={1}\,{\hbox {eV}}$. The broadening is much larger than for other bands because these bands are dispersionless, which makes them more susceptible to disorder and localization as compared, for instance, to the Dirac cone states.

We next study the delocalization of the electronic states and their robustness against disorder. The question of disorder-induced localization is independent of the changes in the bandwidth (see “(Projected) inverse participation ratio” in the Methods section). Based on the clear separation of the $\pi$-system we can calculate the delocalization of the wave functions within the $\pi$-system by means of the inverse participation ratio (IPR)^71^. The IPR is a well-established measure for the spread of wave functions, which has already been used successfully for 2D-Dirac materials and topological insulators^72,73^. It is defined as

4 $$\begin{aligned} \text {IPR}(n) := \sum _{i=1}^N \left| \left\langle \,i\,|\,n, {\varvec{k}}=0\,\right\rangle \right| ^4 , \end{aligned}$$

where the sum runs over all *N* orbitals in a supercell. $|i\rangle$ denotes a WO and $|n,{\varvec{k}}=0\rangle$ is the analyzed eigen-state of the system. To focus on the $\pi$-states only, we utilize here the projected IPR ($\pi$-IPR) defined in the “Methods” section, where $|i\rangle$ is restricted to $\pi$-orbitals. Since the IPR values depend on the number of orbitals and the choice of supercell, we can only compare the phenyl COFs with each other and, separately, the biphenyl COFs with each other.

**Figure 12:**
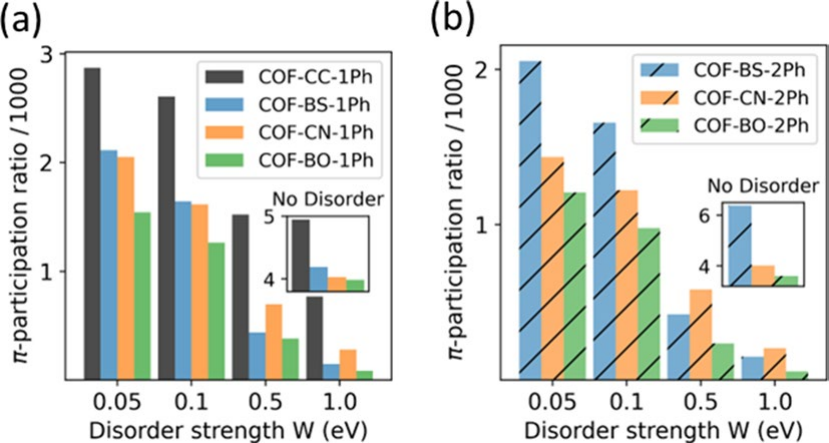
Participation ratio of $\pi$-states for different strength of Anderson disorders in a supercell. Calculations without disorder are shown in the inset for the same supercell.

A more intuitive and closely related quantity is the participation ratio (PR), which is defined as the inverse of the IPR. It can be understood as the average number of orbitals over which a wave function is distributed. Figure 12 shows the average PR that is obtained for the entire set of $\pi$-states and compares the studied 2D COFs. In all cases one observes that the $\pi$-PR decreases with increasing disorder, which induces to a stronger localization of these states. Starting from pristine systems ($W=0$) in which an average $\pi$-state is spread over 32% (COF-BO-1Ph) to 39% (COF-CC-1Ph) of the $\pi$-orbitals, at $W={0.1}\,{\hbox {eV}}$, the average spread is reduced to 20.6% for COF-CC-1Ph and strongly suppressed to 10.0% for COF-BO-1Ph. To put this into perspective, this corresponds to an average (de-)localization over 174 and 84 pores, respectively, which are in the same order of magnitude as experimentally achieved domain sizes^14,74,75^. For the same *W*, electronic states of COF-BS-1Ph and COF-CN-1Ph are (de-)localized on average over 110 and 108 pores, respectively. These values are surprisingly similar and are indeed not expected given the strong differences in the NICS aromaticities of the linker monomers.

For larger strength of disorder ($W\ge {0.5}\,{\hbox {eV}}$) we observe a switch in the order of the PR values between COF-BS-1Ph and COF-CN-1Ph (cf. Fig. 12a) and also for the corresponding biphenyl COFs (cf. Fig. 12b), suggesting that CN-based COFs are more resilient to energetic disorder than BS-based COFs. This behavior can be correlated to the energy-resolved PR values which can vary by more than one order of magnitude for a single systems (not shown as a plot). A detailed analysis of the band- and energy-resolved PR shows strong differences between band edge and band center with more delocalized states towards the middle of the bands for CN-based COFs, especially in the hcb-tri group, which increase the average PR at these energies while analogous states in the middle of the bands of BS-COFs are less delocalized. States at the band edges are much more strongly localized in both systems.

**Figure 13:**
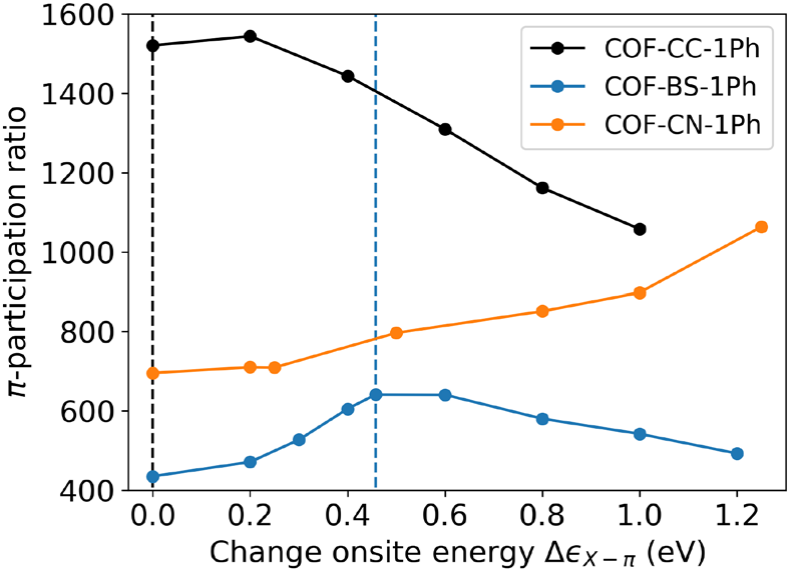
Changes in the delocalization of $\pi$-states (measured by the participation ratio with disorder $W={0.5}\,{\hbox {eV}}$) upon shifting the linker’s onsite-energy. Vertical dashed lines indicate where onsite energies for linker and phenyl rings would coincide, i.e. $|\epsilon _{\text {X-}\pi }+\Delta \epsilon _{\text {X-}\pi } - \epsilon _{\text {C=C-}\pi }|$=0. For COF-CC-1Ph this ocurs at $\Delta \epsilon _{\text {X-}\pi } = {0}\,{\hbox {eV}}$, for COF-BS-1Ph at $\Delta \epsilon _{\text {X-}\pi } = {0.46}\,{\hbox {eV}}$ and for COF-CN-1Ph at $\Delta \epsilon _{\text {X-}\pi } = {2.87}\,{\hbox {eV}}$).

We finally study how the delocalization of electronic states can be increased upon linker-based doping for the assumed disorder regime of $W={0.5}\,{\hbox {eV}}$. Figure 13 shows the changes in the $\pi$-PR for energetic shifts $\Delta \epsilon _{X-\pi }$ of up to ${1.25}\,{\hbox {eV}}$ that are achievable with conventional molecular dopants even in non-porous organic systems^76^. In all cases one observes that the induced energetic differences have a strong impact on the $\pi$-PR. Vanishing energetic differences between linker and phenyl rings $|\epsilon _{\text {X-}\pi } - \epsilon _{\text {C=C-}\pi }|$ (indicated by dashed lines in Fig. 13) can be understood as resonance condition and always lead to much better delocalization. For instance the $\pi$-PR increases up to 47% for COF-BS-1Ph as compared to the undoped case. We therefore confirm that even in cases with large disorder, energetic differences of $\pi$-orbitals between linker and spacer units have a large impact. For small disorder this resonance effect is further amplified.

## Conclusion

In this work, we compared a family of COFs with the same symmetry but different linker units in terms of $\pi$-conjugation, aromaticity and resulting electronic properties which are all routed in the COF’s $\pi$-system. Although the electronic bands (including $\sigma$- and lone-pair-bands) of the COFs may appear quit different at first glance, we found that the $\pi$-bands are very similar in terms of band shape and arrangement. Our results show that the structure and symmetry alone determines the band dispersions qualitatively regardless of the chemistry of the linker unit. The chemical elements on the other hand control, through bond polarizations and aromaticity, the bandwidth and delocalization, quantitatively. However, the impact on single bands (e.g. bands near the Fermi level) might be very different and uncorrelated.

The understanding of electronic properties is facilitated by the transformation of the crystal Bloch states to localized Wannier orbitals with $\sigma$, $\pi$ and lone-pair character. This WO representation of the electronic states resonates well with chemical intuition and is a natural basis for analyzing the $\pi$-system and allows rationalizing simpler effective topological models that are associated to certain subsets of bands. The analysis of electronic bandwidth, robustness against out-of-plane rotations, sensitivity to energetic disorder, delocalization and nuclear independent chemical shifts unveiled chemical trends that are independent of the number of phenyl rings between linkers and only depend on the chemical elements of the linker.

Our study revealed two independent effects of variable $\pi$-conjugation due to differently polarized linker bonds within the studied family. First, 2D COFs with more aromatic linker rings (as measured by NICS) show larger cumulative bandwidths and therefore larger effective TIs between the building blocks. The NICS aromaticity, however, characterizes the $\pi$-system as a whole and is not necessarily a valid descriptor for specific $\pi$-bands, such as the top valence bands. This renders the linker’s aromaticity barely related to (hole) transport properties and transport parameters like effective masses of the top valence bands. A second aspect of $\pi$-conjugation is the delocalization of wave functions within the $\pi$-system. This depends mainly on energetic differences between $\pi$-orbitals at linker and spacer units and is less related to the aromaticity of the linker or cumulative bandwidth of the $\pi$-system. In fact, even non-aromatic BS linkers can lead to stronger delocalization than aromatic triazine linkers for energetic disorder that is not too strong. However, a larger coupling between building blocks (larger effective TIs) makes aromatic linkers more robust against very large disorder strengths. Other measures of aromaticity that are based on delocalization such as the Shannon aromaticity^30^ (see section SI-9 in the SI) show the same trend as the $\pi$-PR, although, one should keep in mind that the Shannon aromaticity also depends on the size of the linker ring and therefore overestimates delocalization in COF-BS-1Ph and COF-BS-2Ph slightly. We note that measures of aromaticity, which are based on different physical or chemical properties, are generally not necessarily fully compatible to each other^77^ which is also observed for 2D COFs.

Finally, we conclude that minimizing energetic differences between linker and spacer units, for instance by doping, increases delocalization to dozens of pores or more, depending on the disorder, which provides an intuitive and elegant way to tailor electronic properties.

## Methods

### Ab-initio DFT simulations and Wannier orbitals

The symmetrized structures are optimized at DFT level of theory using vasp^78,79^ with PBE exchange correlation functionals and PAW pseudo potentials^80,81^. In all calculations a cutoff energy of ${450}\,{\hbox {eV}}$ is used for the plane wave basis set. The relaxation was performed in two steps. First, ionic relaxation for different sizes of the unit cell are used to determine the lattice constants in a $\Gamma$-point only calculation (for Brillouin zone integration). The optimal size of the unit cell was found at the minimum of the ground state energy. This was done for every COF. In the second step a final relaxations of the optimized and symmetrized structures were performed using a $3\times 3\times 1$ Monkhorst-Pack ${\varvec{k}}$-points grid. A subsequent electronic structure calculation using a $\Gamma$-centered $6\times 6\times 1$ Monkhorst-Pack grid is the basis for all further calculations.

We use the wannier90 code^82^ to obtain MLWF for all valence bands. Starting projections are s-orbitals at the mid-bond positions and alternating double and single bonds are assumed according to the Lewis structure. Slightly different starting points, e.g. different orbital shapes or slightly different positions, lead to the same MLWF. Electronic properties like band structures are in perfect agreement with the DFT results. All MLWFs are real-valued within numerical precision, which shows that the optimization correctly finds a global minimum of the spread function^52^. We want to note that the non-uniqueness of the Lewis structure also reflects in a freedom of choice for MLWF, which is determined by the starting guess of the Wannierization. However, alternative configurations are equivalent and give the same results.

In situation, where $\pi$-like and $\sigma$-like orbitals are centered at the same position (double bond), the obtained MLWF from wannier90 are not typical $\pi$- and $\sigma$-orbitals but superpositions $|\pm \rangle =\frac{1}{\sqrt{2}}(|\pi \rangle \pm |\sigma \rangle )$. Performing the inverse transformation (i.e. symmetrization) is easily possible and yields typical $\pi$-like and $\sigma$-like molecular orbitals, where only the $\pi$-like orbitals have contributions from atomic $p_z$-orbitals.

### Nuclear independent chemical shift (NICS)

For the calculation of NICS we use the gaussian16 code^83^ and the B3LYP exchange-correlation functional^84,85^. We created a structure, which contains only one pore of the COF and is saturated with hydrogen at the outermost phenyl rings. With this setting we make sure that no magnetic fields from other ring currents than the central COF-pore and their constituents disturb the result. All atoms lie in the x–y plane and NICS values are calculated ${1}\,{\text{\AA} }$ above the plane. Therefore, only the ZZ-component of the magnetic shielding tensor, which is the negative of $\text {NICS}_{ZZ}(1)$, is of relevance.

### Out-of-plane rotation

To measure the effect of out-of-plane rotations of the phenyl rings we perform separate band-structure calculations for all rotation angles and align them at their vacuum levels, which allows a proper comparison of band energies $E^{(\phi )}_n({\varvec{k}})$.

Furthermore, we define the energy dependent maximal projection as

5 $$\begin{aligned} P^{(\phi )} (E) = \max \limits _{{\varvec{k}}\in \Lambda }\{p^{(\phi )}_{z,\text {linker}} (n,{\varvec{k}})\,|\, E^{(\phi )}_n({\varvec{k}}) = E\}, \end{aligned}$$

where ${\varvec{k}}$ is along the path $\Lambda$ (i.e. $\Gamma$-M-K-$\Gamma$) that is representative for the Brillouin zone. Note that $p_z$-orbitals from the rotated phenyl rings are not included in the projection.

For quantitative results we introduce the $\phi$-dependent *cumulative bandwidth* (CBW) as,

6 $$\begin{aligned} \text {CBW}(\phi ) := \int _{-\infty }^{E_F} dE \, \Theta \left( P^{(\phi )}(E) - P_\text {min} \right) , \end{aligned}$$

similar to the cumulative bandwidth in the main text, where $\Theta (x)$ denotes the Heaviside-step-function and $P_\text {min}$ is the minimum projection required for assigning states to a $\pi$-band. The value can easily be chosen such that individual bandwidths for $\phi =0$ are in agreement with bandwidths in Fig. 6 (main text).

### (Projected) inverse participation ratio

The inverse participation ratio (IPR)^71^ is defined as the second moment of the probability density,

7 $$\begin{aligned} \text {IPR}(n {\varvec{k}}) := \sum _{i=1}^N \left( p_i^{(n {\varvec{k}})} \right) ^2 , \end{aligned}$$

where the sum runs over all *N* orbitals in a unit cell and $p_i^{(n {\varvec{k}})}$ is the occupation probability of the orbital *i* for the state $|n {\varvec{k}}\rangle$ given by

8 $$\begin{aligned} p_i^{(n {\varvec{k}})} := \left| \left\langle \,i\,|\,n {\varvec{k}}\,\right\rangle \right| ^2 . \end{aligned}$$

Since we are first of all interested in the delocalization with respect to $\pi$-orbitals we introduce the projected IPR that measures only the delocalization within a certain subsystem $S \subseteq \text {WO}$ (e.g. all $\pi$-orbitals), but is insensitive to all other contributions (e.g. from $\sigma$-orbitals). The projected *S*-IPR is defined in analogy to the general IPR with (renormalized) probabilities ${\tilde{p}}_i^{(n{\varvec{k}})}$

9 $$\begin{aligned} S\text {-IPR}(n{\varvec{k}}) = \sum _{i \in S} \left( {\tilde{p}}_i^{(n{\varvec{k}})} \right) ^2, \end{aligned}$$

with

10 $$\begin{aligned} {\tilde{p}}_i^{(n{\varvec{k}})} = \frac{p_i^{(n {\varvec{k}})}}{\sum _{j \in S} p_j^{(n {\varvec{k}})}} . \end{aligned}$$

${\tilde{p}}_i^{(n{\varvec{k}})}$ is the normalized projected probability with $\sum _{i \in S} {\tilde{p}}_i^{(n{\varvec{k}})} = 1$.

For the actual calculation we create a real-space $29\times 29\times 1$ supercell for 1Ph COFs and a $23\times 23\times 1$ supercell for 2Ph COFs and add Anderson onsite disorder. The disorder is generated from a uniform distribution of random numbers in the interval $[-W/2, W/2]$, where *W* is the strength of the disorder. A direct diagonalization using the lapack library^86^ is performed to get eigenstates and eigen-energies at the $\Gamma$-point of the supercell. The (projected) IPR are calculated for every eigenstate and inversion provides the participation ratio for every state. The results are independent of the actual realizations of random numbers, which we tested by applying different seeds of the random number generator. Successful examples, where the IPR has been used in conjunction with Anderson disorder can be found in the literature^87^.

## Supplementary Information

Supplementary Information.

## Data Availability

The data that support the findings of this study are available from the corresponding author upon reasonable request.
