# Supplementary material for: Understanding the electronic pi-system of 2D covalent organic frameworks with Wannier functions
Source: Sci Rep. 2023 Jan 30;13:1685. doi: 10.1038/s41598-023-28285-w (PMC9886956; doi:10.1038/s41598-023-28285-w)
Supplement: Supplementary file 1 — Supplementary Information. [file 41598_2023_28285_MOESM1_ESM.pdf]

# – Supporting Information – Understanding the electronic pi-system of 2D covalent organic frameworks with Wannier functions

Konrad Merkel, Johannes Greiner and Frank Ortmann*
TUM School of Natural Sciences, Technical University of Munich

September 27, 2022

SI-1 Quality of Wannierization

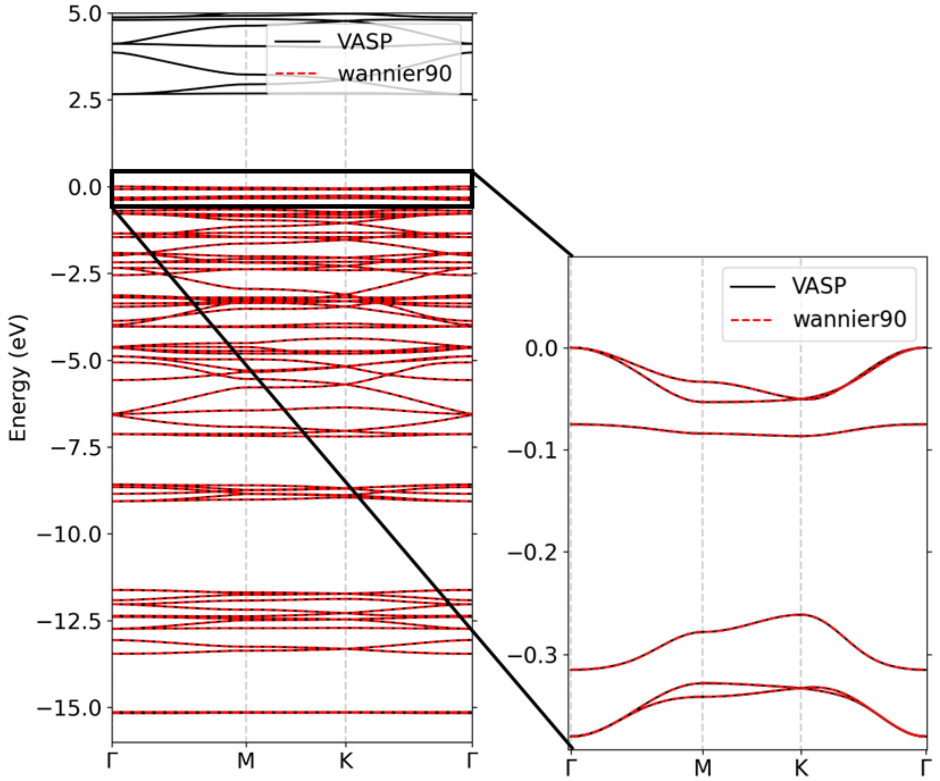

Figure SI-1: Comparison between band structure before the wannierization (VASP) and after wannierization (wannier90).

SI-2 Orbitals and Transfer Integrals

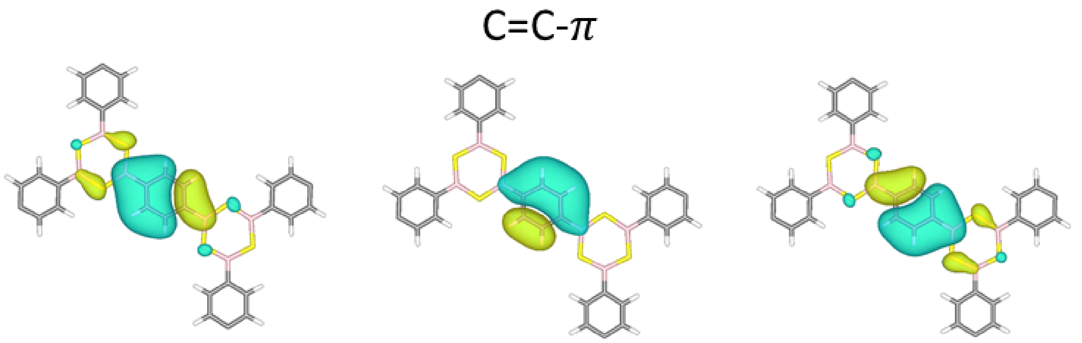

Figure SI-2: All C=C-π orbitals of a single phenyl ring. Shapes are similar but small deformations occur in the vicinity of the linker.

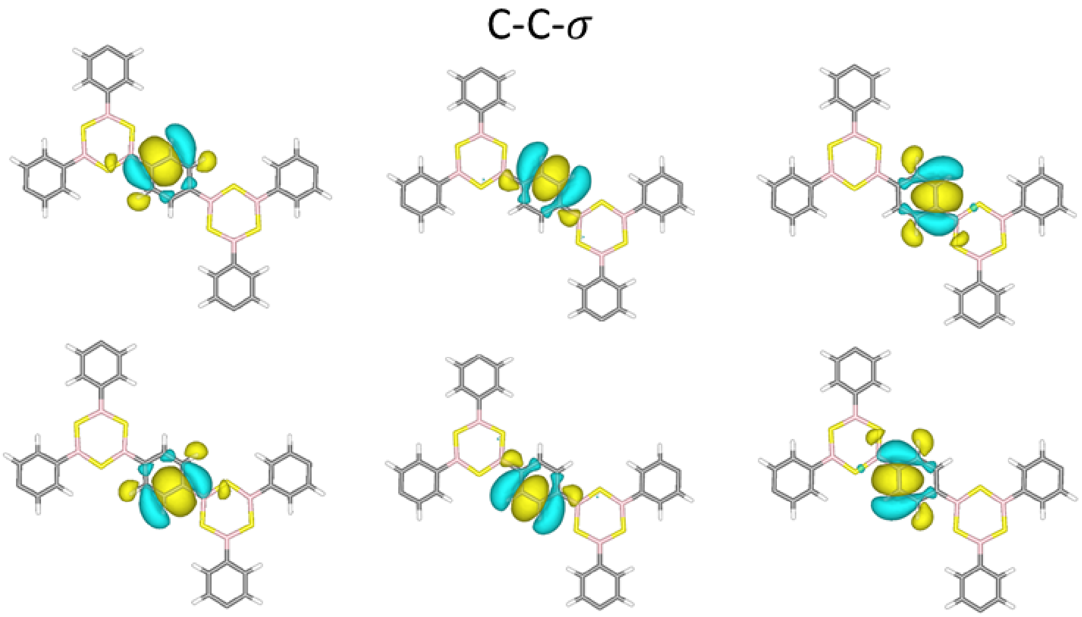

Figure SI-3: All C-C-σ orbitals of a single phenyl ring. Shapes are similar but small deformations occur in the vicinity of the linker. C-C-σ orbitals at single and double bond positions (C-C-σ_s, C-C-σ_d) have the same shape.

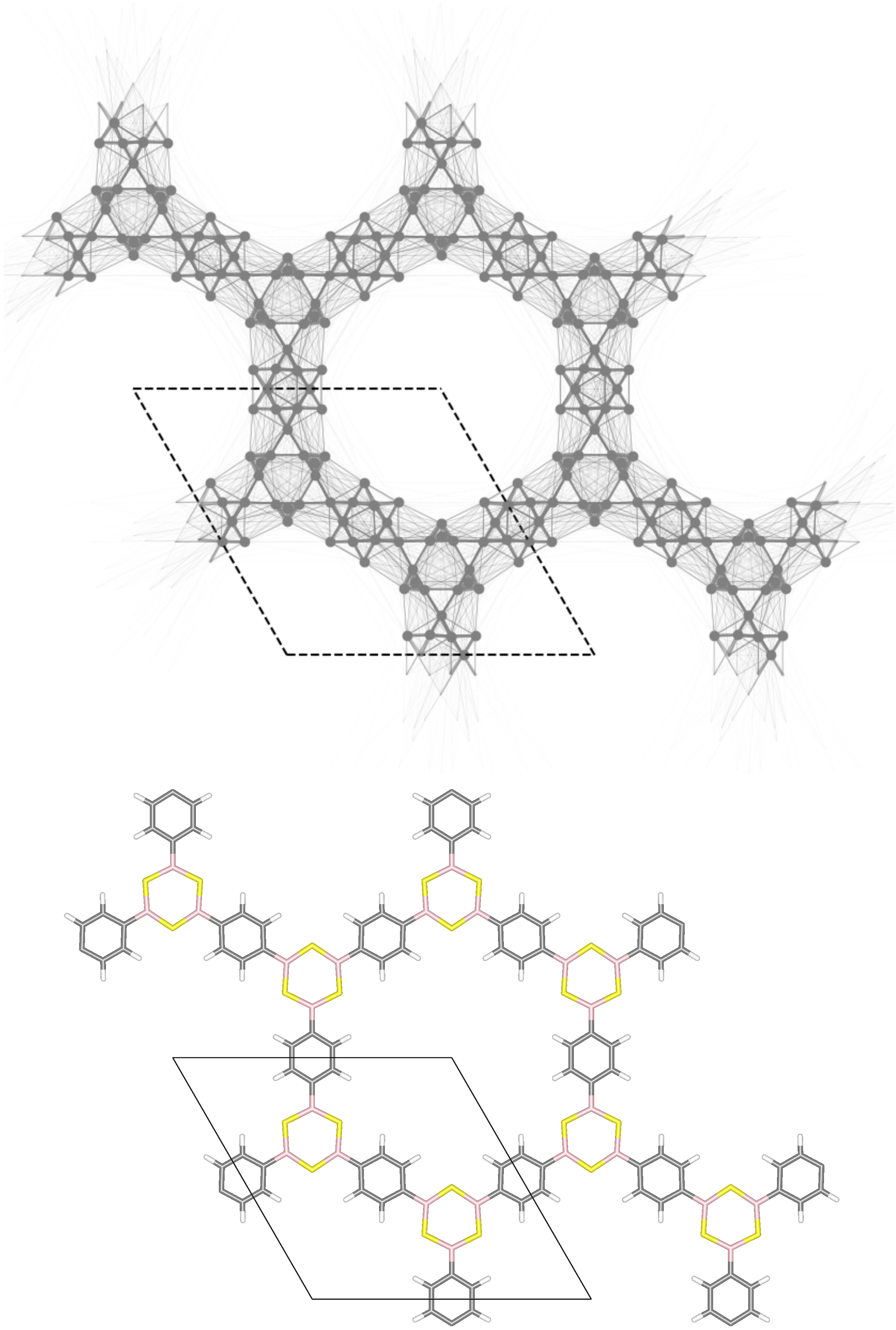

Figure SI-4: Map of transfer integrals (TI) larger than 1 meV for COF-BS-1Ph. Dots represent the center of every Wannier orbital, straight lines show the corresponding TI between orbitals, where the line width characterizes the value of the TI. The unit cell is highlighted with dashed lines. The lower panel shows the corresponding COF-pore.

(a) COF-BO-1Ph

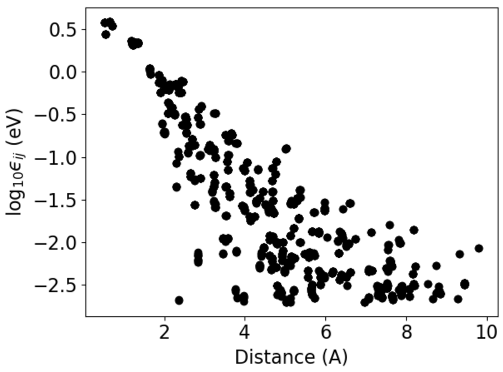

(b) COF-BO-2Ph

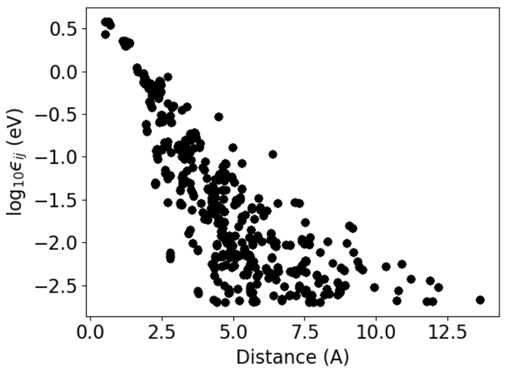

(c) COF-BS-1Ph

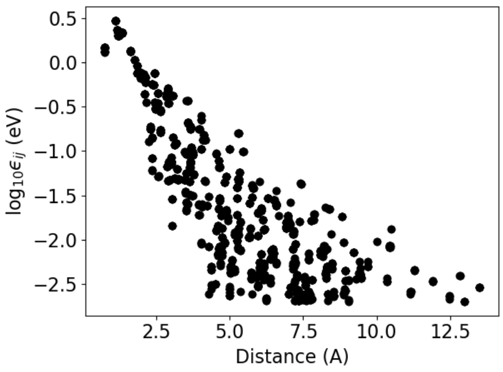

(d) COF-BS-2Ph

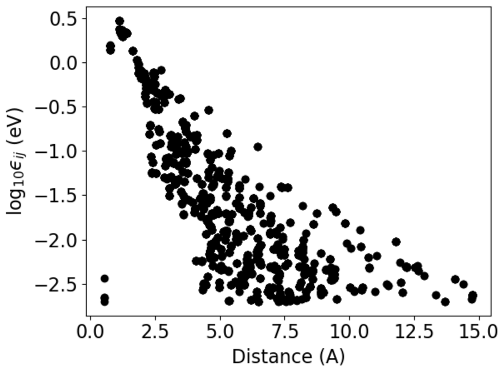

(e) COF-CN-1Ph

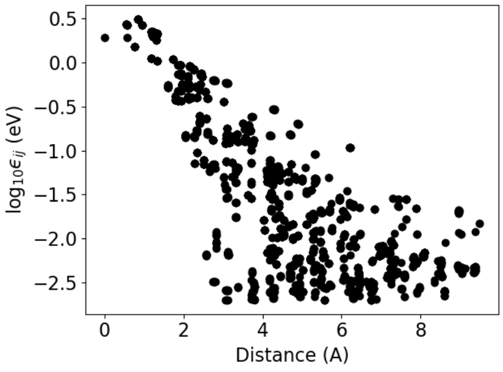

(f) COF-CN-2Ph

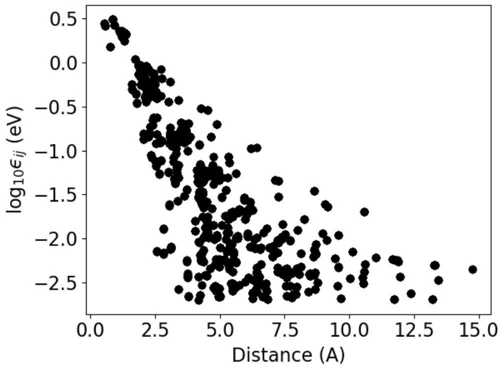

(g) COF-CC-1Ph

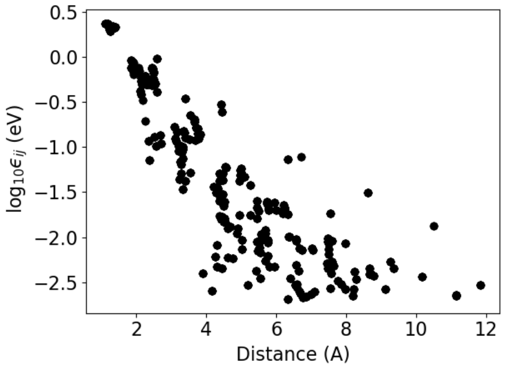

Figure SI-5: Values of Transfer integrals (log) and their distance. All figures show that TI become exponentially suppressed for large distances.

SI-3 Band Structure

The subsequent figures show the valence band structures and their projection onto the π-system for the investigated COFs. Systematic investigations of the band gap for COF-BO-1Ph, COF-BO-2Ph, COF-CN-1Ph and COF-CN-2Ph can be found in Ref.[1].

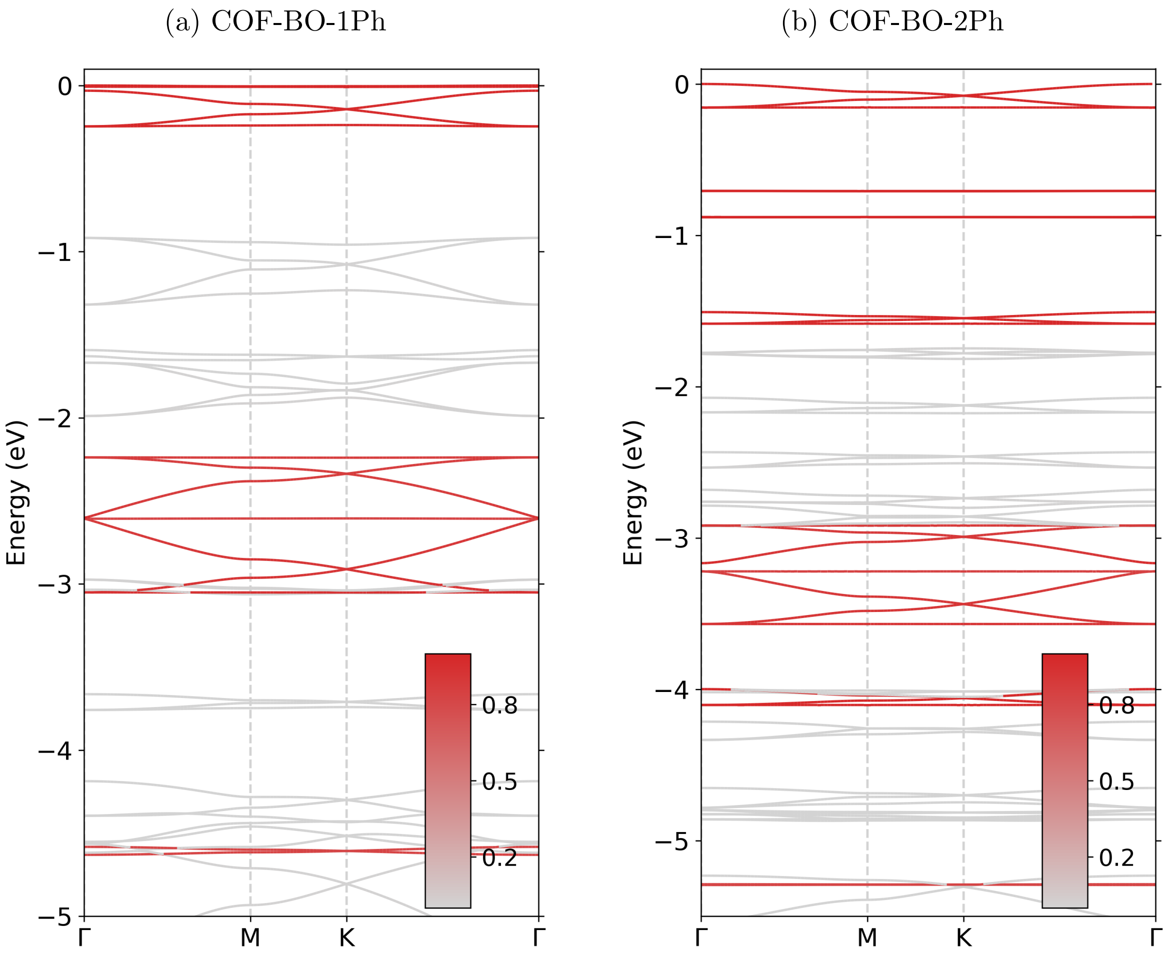

Figure SI-6: Band structures for BO-COFs

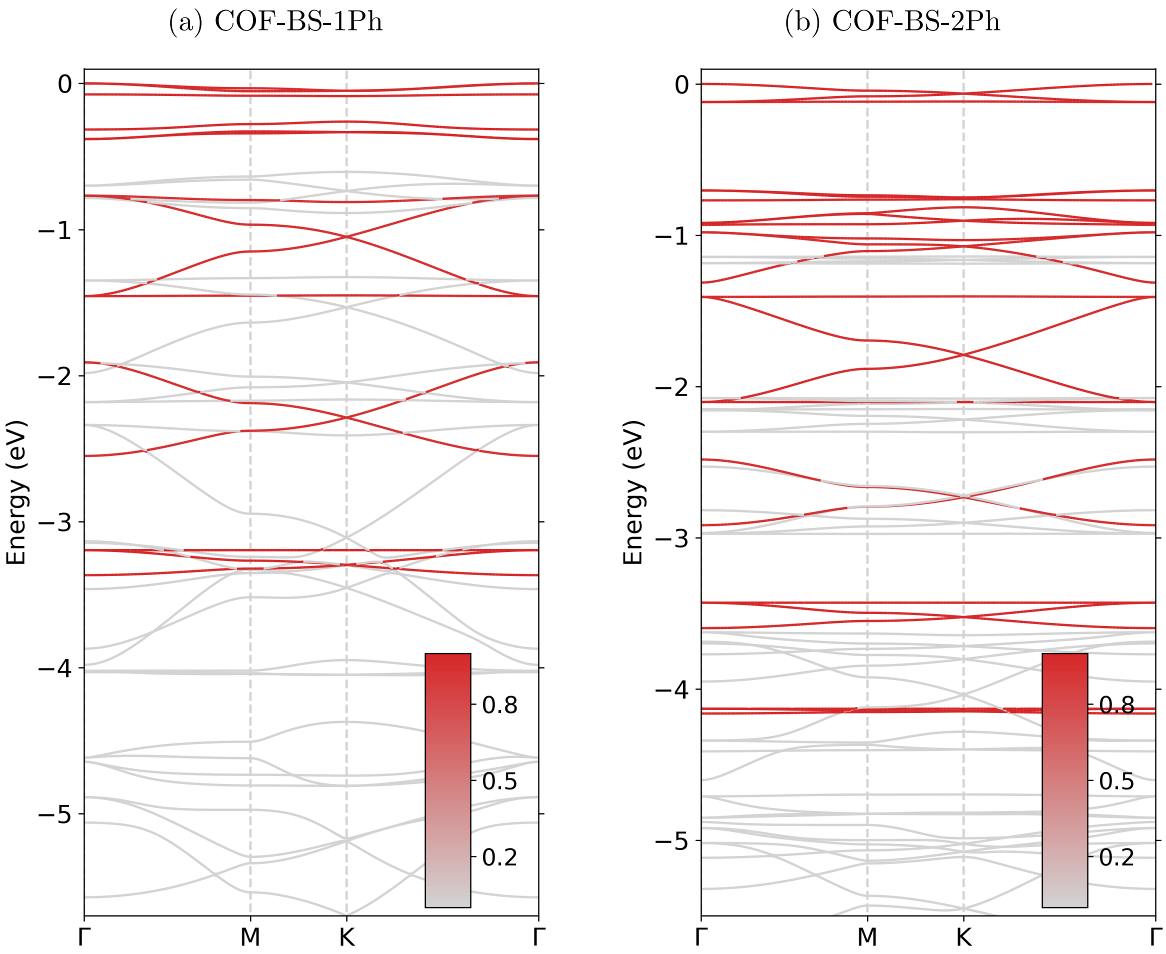

Figure SI-7: Band structures for BS-COFs

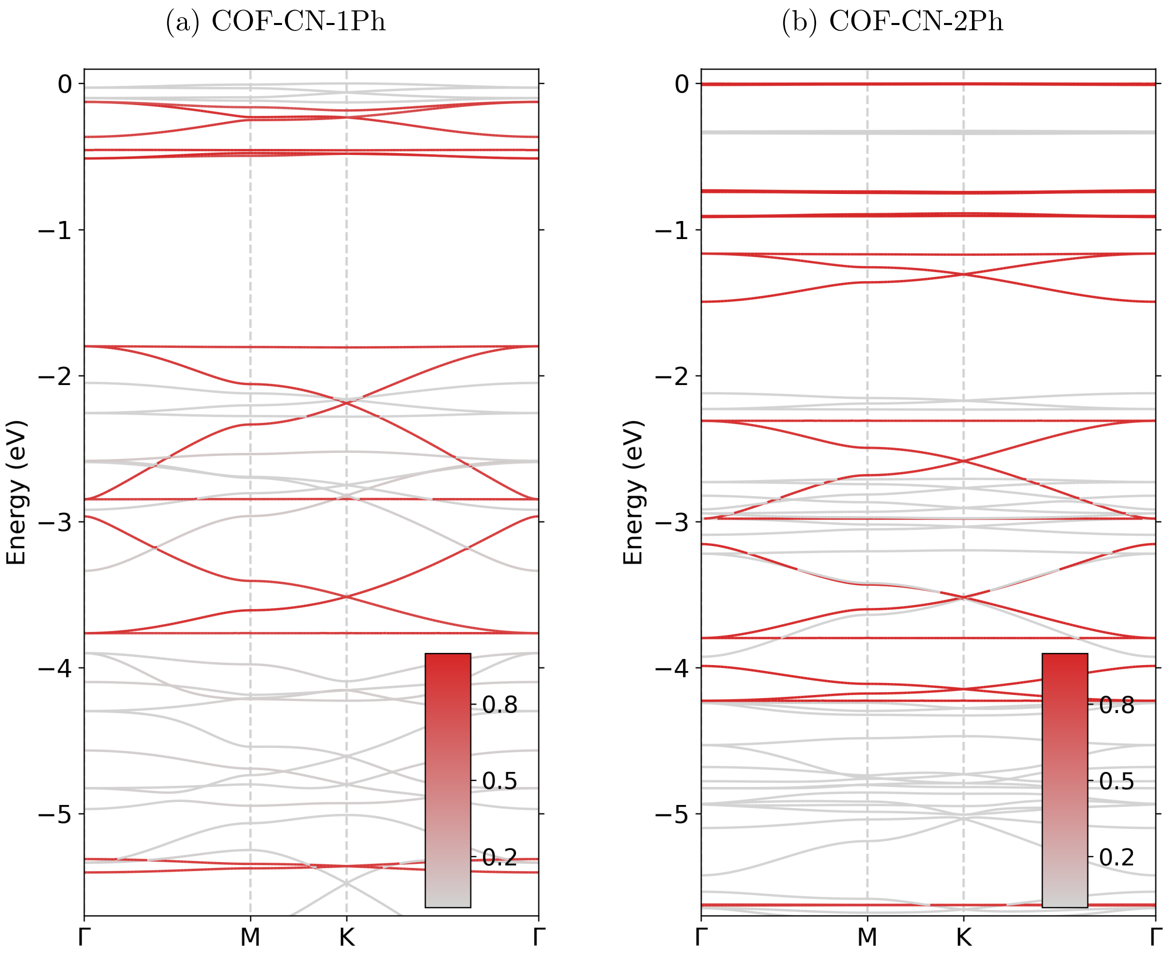

Figure SI-8: Band structures for CN-COFs

(a) COF-CC-1Ph

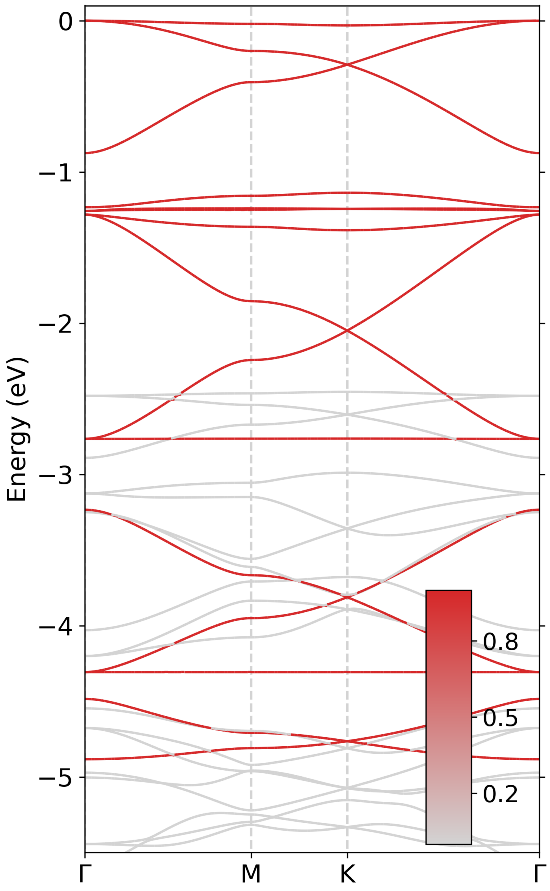

Figure SI-9: Band structure for reference COF

Table SI-1: Bandwidth of 1Ph COFs in eV

Band group	COF-CC-1Ph	COF-BS-1Ph	COF-CN-1Ph	COF-BO-1Ph
1	0.875	0.075	0.24	0.008
2	0.026	0.065	0.058	0.215
3	1.483	0.675	1.048	0.363
4	1.074	0.641	0.800	0.443
5	0.399	0.171	0.091	0.048

Table SI-2: Bandwidth of 2Ph COFs in eV

Band group	COF-BS-2Ph	COF-CN-2Ph	COF-BO-2Ph
1	0.12	0.008	0.156
2	0.066	0.011	0.0003
3	0.013	0.007	0.001
4	0.333	0.33	0.077
5	0.695	0.669	0.249
6	0.434	0.644	0.348
7	0.168	0.238	0.105
8	0.032	0.006	0.005

SI-4 Bader Charge

Bader charges[2,3] are calculated for the ground state using the bader charge analysis code[4,5]. DFT calculations are described in the methods section of the main text. Results are shown in Tab. SI-3.

Table SI-3: Bader charge of linker species for all COFs.

COF	Linker Atom 1 (X=C,B) [C]	Linker Atom 2 (Y=S,N,O,C) [C]
COF-CC-1Ph	4.1	3.9
COF-BS-1Ph	1.3	7.1
COF-BS-2Ph	1.3	7.1
COF-CN-1Ph	3.0	6.1
COF-CN-2Ph	2.9	6.2
COF-BO-1Ph	0.8	7.5
COF-BO-2Ph	0.8	7.5

SI-5 Effective Mass

Effective masses are obtained in the real space basis of MLWF as described in Ref.[6]. $\boldsymbol{k}$-derivatives of the Fourier series (c.f. Eq. (1) in the main text) can be performed analytically, where the only difficulty is the calculation of $\nabla_{\boldsymbol{k}} U_{nm}(\boldsymbol{k})$, which can be obtained by $\boldsymbol{k} \cdot \boldsymbol{p}$ perturbation theory[6,7]. The obtained effective masses in Tab. SI-4 show better or equally good agreement than direct fits of the band structure, which are always dependent on finite $\boldsymbol{k}$-differences. Degenerate bands are denoted with * or †. Components of m^* are given with respect to reciprocal lattice vectors. The eigenvalues of m^* are given in the last two columns. In general the effective mass tensors for different bands do not share the same eigensystem.

Bands near the Fermi level are very flat for all COFs except for COF-CC-1Ph ($\Delta E = 0.88\,\mathrm{eV}$). The resulting effective masses are therefore very high. Flat bands (from kgm bands) only have finite effective masses due to distortions.

The highest occupied bands are π-bands, except for COF-CN-1Ph, where the highest occupied bands originate from lone-pair (lp) orbitals at the linker and are very flat.

Table SI-4: Effective mass tensor m^* at Γ-point expressed in reciprocal basis for uppermost group of bands in units of electron rest mass m_e. Degeneracies at Γ-point among a group are marked with * or †. The underlying orbitals that correspond to the a band are given in brackets (π or lp). The effective mass tensor is symmetric $m^*_{12} = m^*_{21}$. Last two columns contain the eigenvalues of the effective mass tensor.

Material	Band	m^*_{11} (m_e)	m^*_{22} (m_e)	m^*_{12} (m_e)	Eig.val. 1 (m_e)	Eig.val. 2 (m_e)
COF-CC-1Ph	HOMO* (π)	-37.9	-42.6	35.7	-4.4	-76.1
COF-CC-1Ph	HOMO-1* (π)	-21.7	-17.1	-5.9	-25.7	-13.0
COF-CC-1Ph	HOMO-2 (π)	1.5	1.5	-0.8	2.3	0.8
COF-BS-1Ph	HOMO* (π)	-27.8	-28.1	16.2	-11.7	-44.2
COF-BS-1Ph	HOMO-1* (π)	-25.1	-24.8	10.3	-35.2	-14.7
COF-BS-1Ph	HOMO-2 (π)	-257.9	-257.7	135.1	-392.9	-122.7
COF-CN-1Ph	HOMO* (lp)	11.4	-322.3	79.7	29.4	-340.4
COF-CN-1Ph	HOMO-1* (lp)	-291.8	15.2	68.0	-306.2	29.6
COF-CN-1Ph	HOMO-2† (lp)	160.1	131.9	-166.9	313.4	-21.5
COF-CN-1Ph	HOMO-3† (lp)	8.1	33.8	83.8	-63.9	105.7
COF-CN-1Ph	HOMO-4* (π)	-26.1	-19.9	7.7	-31.3	-14.6
COF-CN-1Ph	HOMO-5* (π)	-24.9	-30.9	17.7	-9.9	-45.9
COF-CN-1Ph	HOMO-6 (π)	16.8	16.8	-8.4	8.4	25.2
COF-BO-1Ph	HOMO* (π)	-237.5	-193.3	103.9	-321.6	-109.1
COF-BO-1Ph	HOMO-1* (π)	-194.6	-239.2	112.2	-102.5	-331.3
COF-BO-1Ph	HOMO-2 (π)	-8406.2	4061.0	-13477.5	-17021.9	12676.7
COF-BS-2Ph	HOMO (π)	-33.8	-33.7	16.8	-50.6	-16.9
COF-BS-2Ph	HOMO-1* (π)	209.5	87.9	64.1	237.0	60.3
COF-BS-2Ph	HOMO-2* (π)	269.8	388.3	-301.2	22.1	636.0
COF-CN-2Ph	HOMO* (π)	7205.5	1747.0	2510.9	8184.8	767.7
COF-CN-2Ph	HOMO-1* (π)	-7256.0	-11618.3	9476.5	287.0	-19161.4
COF-CN-2Ph	HOMO-2 (π)	292.8	304.6	-159.3	139.3	458.1
COF-BO-2Ph	HOMO-0 (π)	-29.7	-29.8	14.9	-14.9	-44.6
COF-BO-2Ph	HOMO-1* (π)	1005.1	1724.6	1271.7	43.3	2686.4
COF-BO-2Ph	HOMO-2* (π)	3611.2	3015.1	-3285.0	6611.6	14.7

Similar states also exist in COF-CN-2Ph but with lower energy, which makes them unimportant for transport. For COF-CN-1Ph we have given the top nine bands to also provide information about the top bands of the π-system.

SI-6 Onsite energy

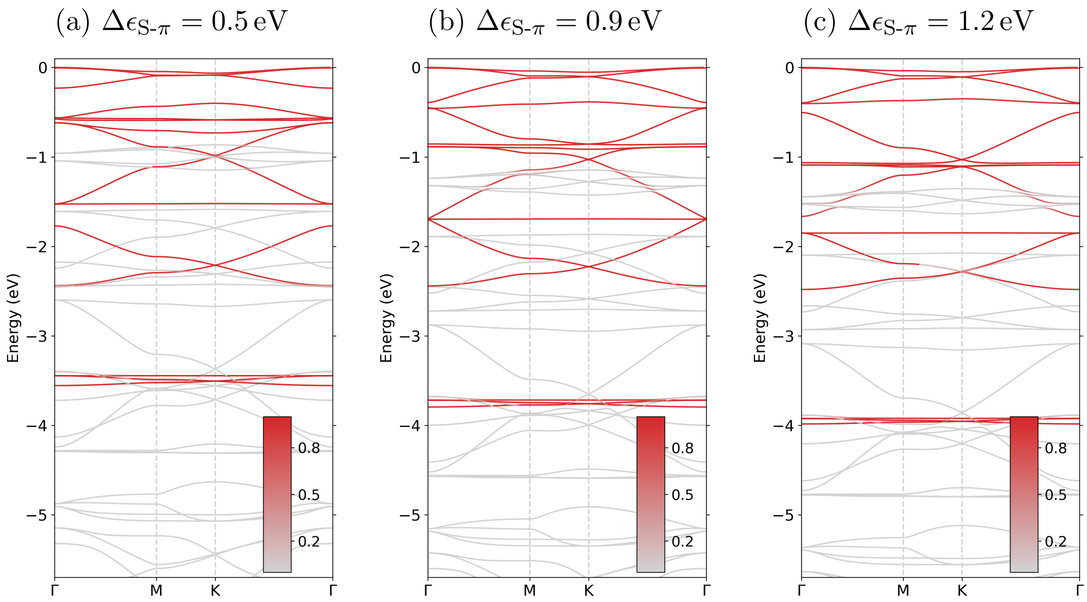

Figure SI-10: Band structure of COF-BS-1Ph for different changes of S-π onsite energy. Fermi energy is chosen individually to be the valence band maximum in each plot.

SI-7 Robustness and Breaking of π-Conjugation by Bond Torsion

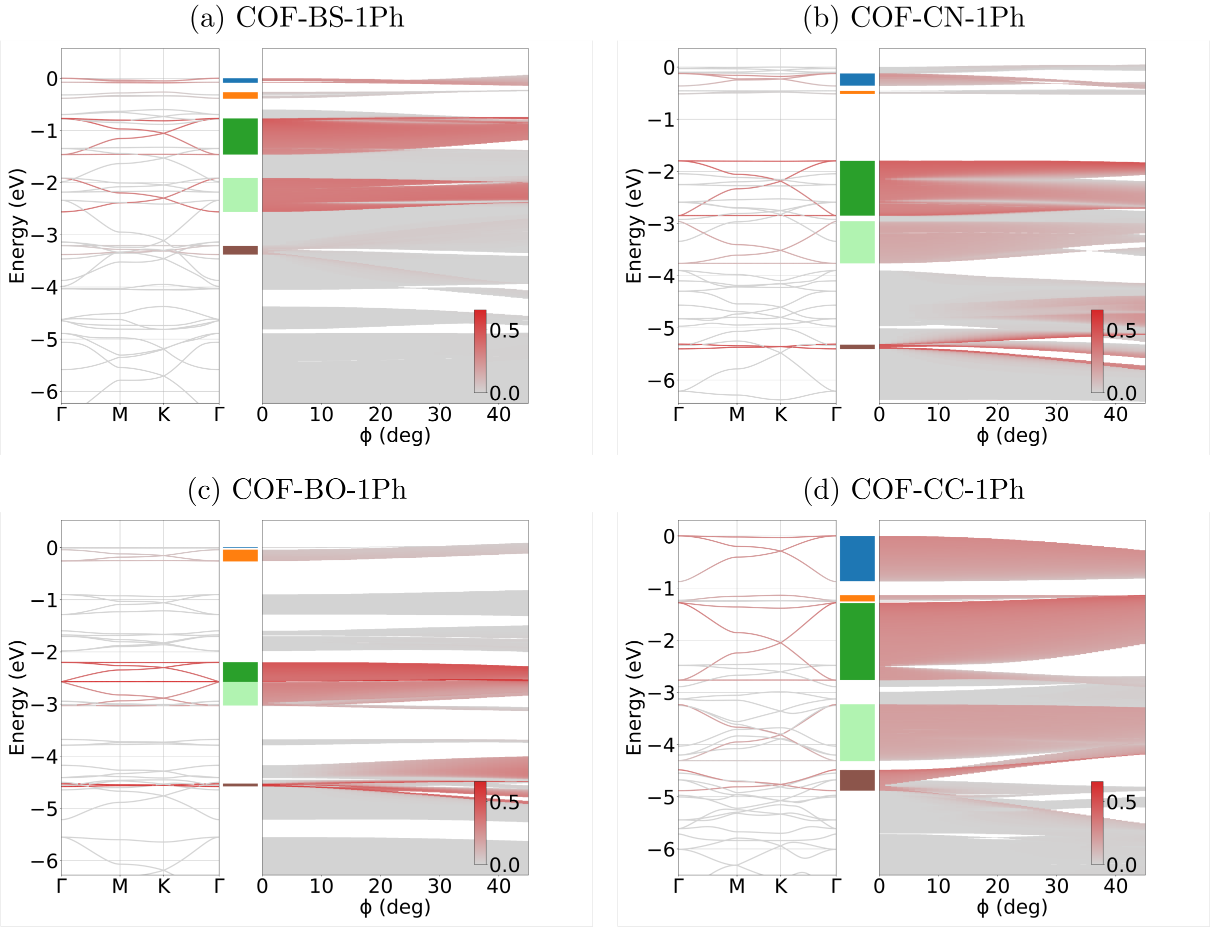

Figure SI-11: Band structure for one phenyl systems (including projection on p_z-orbitals at linker positions) and impact of rotation of the phenyl rings on band width and projection. The energy zero is set to the valence band maximum of the planar structure.

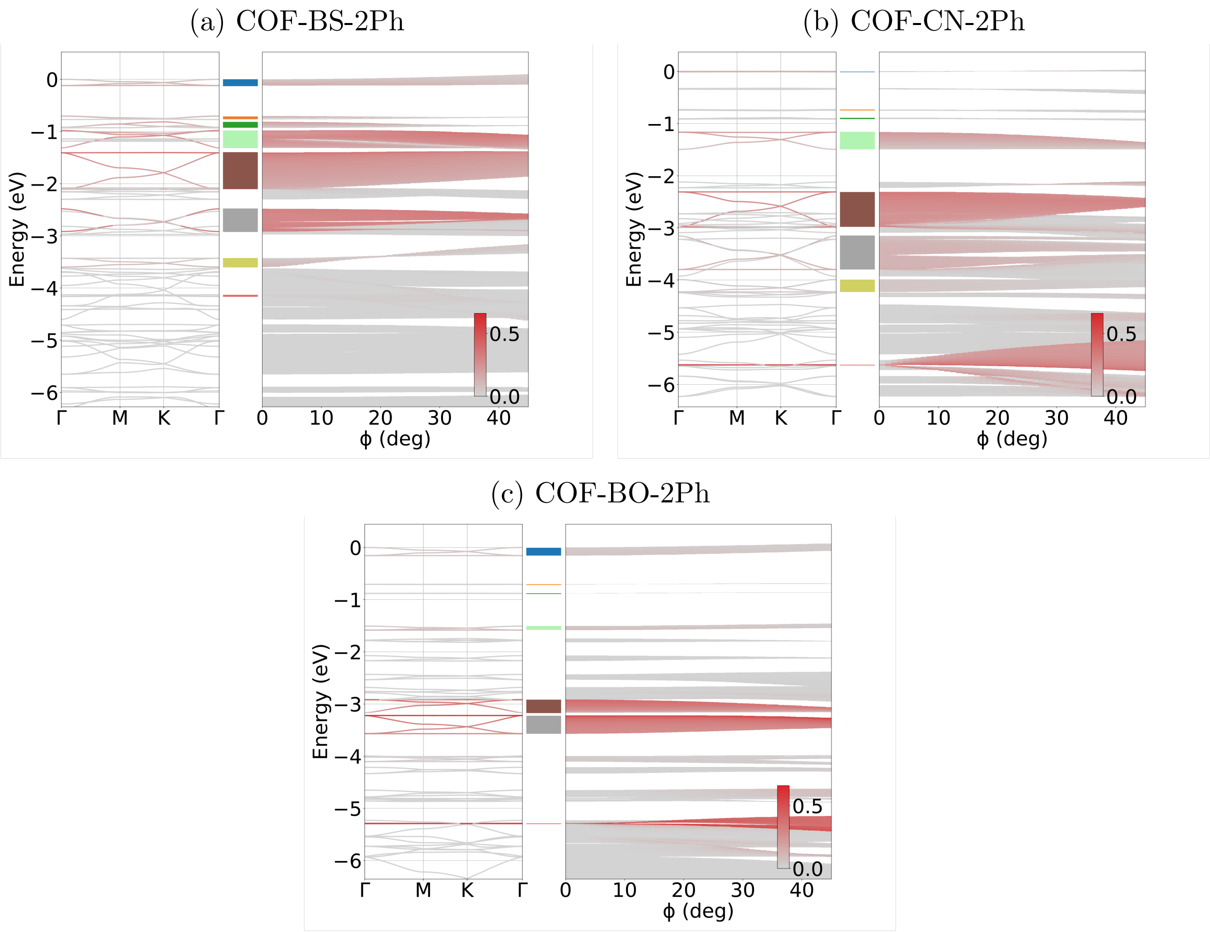

Figure SI-12: Band structure for two phenyl systems (including projection on p_z-orbitals at linker positions) and impact of rotation of the phenyl rings on band width and projection. The energy zero is set to the valence band maximum of the planar structure.

To elaborate the changes of the electronic structure we extend our analysis by comparing our results with the *projected norm* (PN) of $P^{(\phi)}(E)$, which is defined as,

$$PN(\phi) := \int_{-\infty}^{E_F} dE \, \left|P^{(\phi)}(E)\right|^2 . \tag{SI-1}$$

In contrast to CBW (see main text for definition) PN does not depend on any adjustable threshold parameter and is therefore more sensitive to small changes in $P^{(\phi)}(E)$. Including the actual values of $P^{(\phi)}(E)$ makes $PN(\phi)$ also sensitive to possible redistribution of charge density and localization like we have observed for the top kgm-bands near the Fermi-level, which relocate their charge density at the linker completely into p_x and p_y-like orbitals for large ϕ. It is therefore not surprising that $PN(\phi)$ highlights the decline of global π-conjugation even more than $CBW(\phi)$, as can be seen in Fig. SI-13(e)-(f).

In particular, $PN(\phi)$ of COF-CN-1Ph decreases faster than $PN(\phi)$ of COF-CC-1Ph, whereas for CBW it is vise versa. This effect is caused not only by the decrease in projection, but also by gaps in the third π-band (dark green), which are caused by symmetry breaking. COF-CN-1Ph and COF-CN-2Ph show the largest decline. They are therefore more sensitive to rotation as COF-BS-1Ph and COF-BS-2Ph as well as COF-CC-1Ph.

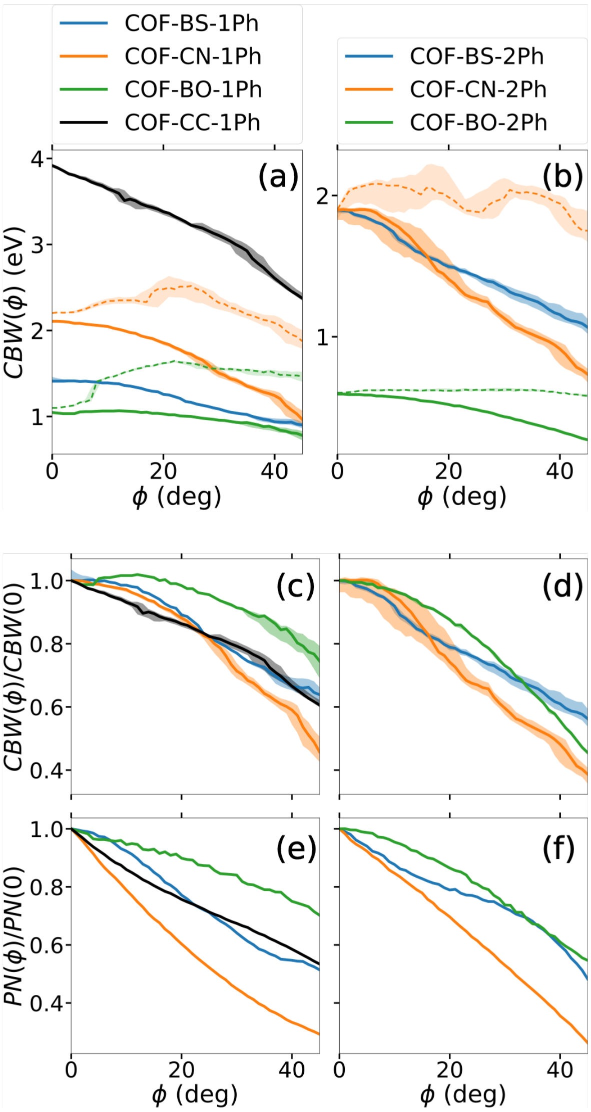

Figure SI-13: Quantitative measures that characterize the influence of rotations. (a)-(b) Cumulative bandwidth $CBW(\phi)$. (c)-(d) Normed cumulative bandwidth $CBW(\phi)/CBW(0)$. (e)-(f) Normed potential norm $PN(\phi)/PN(0)$. All solid lines focus on the upper *pi*-groups (near the Fermi-level); the dashed lines in (a)-(b) show the measurement for all π-groups. The colored areas indicate the deviations at a 15% change of P_{min}.

An initial increase in $PN(\phi)$ is observed for COF-BO-1Ph. This is also caused by a gap in a π-band due to symmetry breaking.

For all other COFs $PN(\phi)$ behaves very similarly to $CBW(\phi)$, which shows that the linker projection only changes merely upon rotation and further justifies our choice of $P_{\min}$ for $CBW(\phi)$.

SI-8 NICS

Table SI-5: Upper bounds of NICS values that originate from ring currents. All values are smaller than numerical precision of the calculation independent of the partition scheme.

COF	NICS (ppm)	Percentage of entire NICS value from COF-pore
COF-CC-1Ph	-0.13	-3.7%
COF-BS-1Ph	0.026	0.8%
COF-CN-1Ph	0.006	0.1%
COF-BO-1Ph	0.024	0.7%

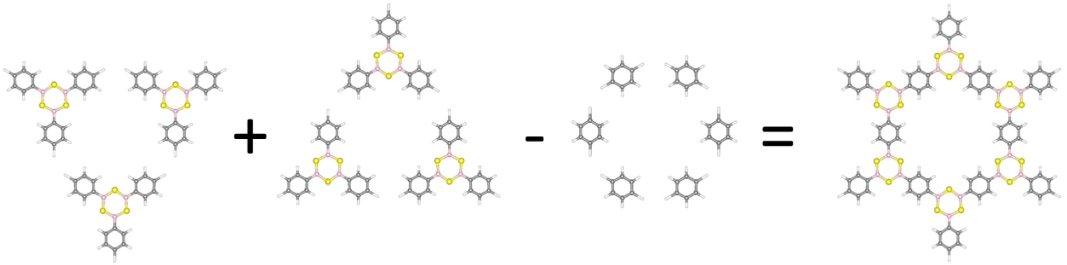

Figure SI-14: Alternative partition scheme for calculating the ring current contribution to NICS. Ring current contribution of NICS was found to be smaller than 0.03ppm, which is smaller than numerical precision for every COF.

SI-9 Shannon Aromaticity

The Shannon aromaticity[8] is defined as

$$S = -\int dr\, \rho(\boldsymbol{r}) \ln \rho(\boldsymbol{r}), \tag{SI-2}$$

which is the Shannon entropy[9] of the ground state charge density $\rho(\boldsymbol{r})$. Values for all COFs are shown in Fig. SI-15. Please note that 1Ph COFs and 2Ph COFs cannot be compared directly due to their different sizes of the unit cell.

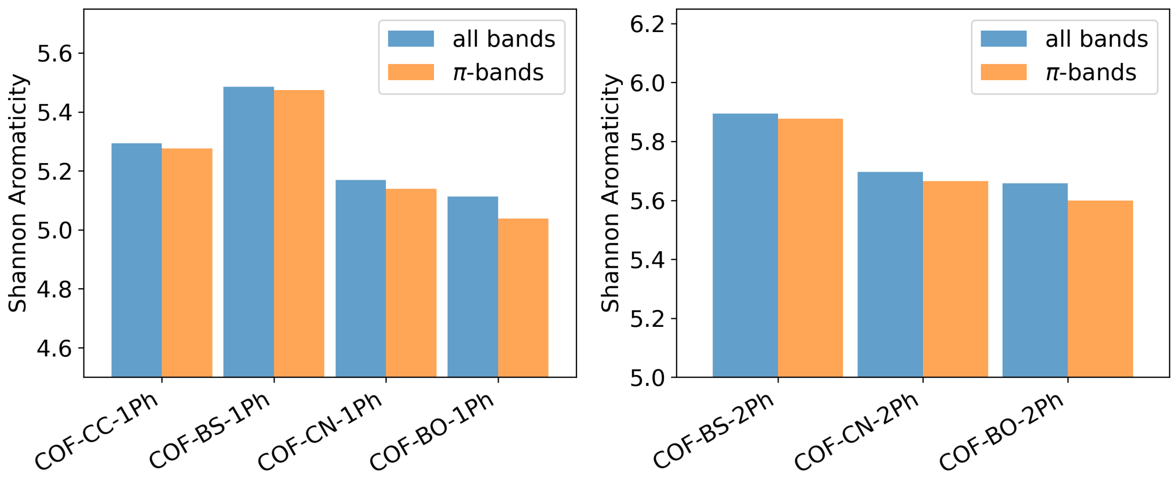

Figure SI-15: Shannon aromaticity

References

[1] Zhu, P. & Meunier, V. Electronic properties of two-dimensional covalent organic frameworks. *J. Chem. Phys.* **137**, 244703 (2012).

[2] Bader, R. F. W. A quantum theory of molecular structure and its applications. *Chem. Rev.* **91**, 893–928 (1991). URL `https://doi.org/10.1021/cr00005a013`. `https://doi.org/10.1021/cr00005a013`.

[3] Bader, R. F. W. The quantum mechanical basis of conceptual chemistry. *Monatsh. Chem.* **136**, 819–854 (2005). URL `https://doi.org/10.1007/s00706-005-0307-x`.

[4] Henkelman, G., Arnaldsson, A. & Jónsson, H. A fast and robust algorithm for bader decomposition of charge density. *Comput. Mater. Sci.* **36**, 354–360 (2006). URL `https://www.sciencedirect.com/science/article/pii/S0927025605001849`.

[5] Yu, M. & Trinkle, D. R. Accurate and efficient algorithm for bader charge integration. *J. Chem. Phys.* **134**, 064111 (2011). URL `https://doi.org/10.1063/1.3553716`. `https://doi.org/10.1063/1.3553716`.

[6] Yates, J. R., Wang, X., Vanderbilt, D. & Souza, I. Spectral and fermi surface properties from wannier interpolation. *Phys. Rev. B* **75**, 195121 (2007).

[7] Wang, X., Yates, J. R., Souza, I. & Vanderbilt, D. Ab initio calculation of the anomalous hall conductivity by wannier interpolation. *Phys. Rev. B* **74**, 195118 (2006).

[8] Yu, D. *et al.* Aromaticity and antiaromaticity of substituted fulvene derivatives: perspectives from the information-theoretic approach in density functional reactivity theory. *Phys. Chem. Chem. Phys.* **19**, 18635–18645 (2017).

[9] Shannon, C. E. A mathematical theory of communication. *Bell Syst. Tech. J.* **27**, 379–423 (1948).
